# Kooperationen zwischen Wissenschaft und Praxis im Öffentlichen Gesundheitsdienst: Ein systematisches Mapping von 2015 bis 2024

**DOI:** 10.1007/s00103-025-04161-y

**Published:** 2025-12-01

**Authors:** Simon Bimczok, Marlene Lakemann, Dagmar Starke, Laura Arnold

**Affiliations:** 1 Akademie für Öffentliches Gesundheitswesen, Düsseldorf (Nordrhein-Westfalen), Düsseldorf, Deutschland; 2 Institut für Medizinische Soziologie, Centre for Health and Society, Medizinische Fakultät und Universitätsklinikum, Heinrich-Heine-Universität Düsseldorf, Düsseldorf, Nordrhein-Westfalen Deutschland

**Keywords:** Öffentliches Gesundheitswesen, Public Health, Aus‑, Fort- und Weiterbildung, Evidenzbasierte Praxis, Wissenstransfer, Public health administration, Public health, Education, public health professional, Evidence-based practice, Translational science, Biomedical

## Abstract

**Hintergrund:**

Wissenschaft-Praxis-Kooperationen im Öffentlichen Gesundheitsdienst (ÖGD) bieten das Potenzial, wissenschaftliche Erkenntnisse in die Praxis und praxisbezogene Fragestellungen in die Forschung zu integrieren. Bestehende Kooperationen können Einblicke in strukturelle Rahmenbedingungen liefern. Bislang existiert in Deutschland jedoch keine systematische Erhebung. Ziel dieser Studie war es daher, Wissenschaft-Praxis-Kooperationen im ÖGD systematisch zu identifizieren und zu analysieren.

**Methodik:**

In einem strukturierten Screeningprozess wurden verschiedene Datenquellen aus dem Zeitraum von 2015–2024 systematisch durchsucht: (i) Google™, (ii) Kongress-Abstract-Bände, (iii) wissenschaftliche Datenbanken, (iv) Datenbanken für graue Literatur, ergänzt um eine (v) Online-Umfrage, ein (vi) Snowballing und (vii) den Einbezug des Netzwerks des EvidenzÖGD-Projektkonsortiums. Die identifizierten Kooperationen wurden anhand verschiedener Strukturmerkmale kategorisiert und deskriptiv mittels nach Subgruppen aufgeschlüsselter Häufigkeitsanalysen ausgewertet.

**Ergebnisse:**

Es wurden insgesamt 611 Wissenschaft-Praxis-Kooperationen im ÖGD identifiziert – vor allem über Kongressbeiträge. Im betrachteten Zeitraum zeigt sich eine Zunahme an Kooperationen. Viele bezogen sich thematisch auf klassische ÖGD-Handlungsfelder und wurden meist auf kommunaler Ebene umgesetzt. Die Koordination war zwischen wissenschaftlichen und ÖGD-Institutionen fast gleich verteilt, während die geografische Umsetzung durch westliche Flächenländer geprägt war.

**Fazit:**

Diese Übersicht über Wissenschaft-Praxis-Kooperationen ermöglicht umfassende Einblicke in eine komplexe und vielschichtige Kooperationslandschaft. Aufbauend bietet sich viel Potenzial für tiefergehende Analysen, z. B. zur Untersuchung förderlicher wie hinderlicher Faktoren der Zusammenarbeit sowie zum besseren Verständnis räumlicher und thematischer Cluster.

**Zusatzmaterial online:**

Zusätzliche Informationen sind in der Online-Version dieses Artikels (10.1007/s00103-025-04161-y) enthalten.

## Abstract (en)

**Background:**

Collaboration between science and practice in the public health service (PHS) holds the potential to translate scientific findings into practice and to incorporate practice-oriented questions back into research. Existing collaborations can provide valuable insights into structural conditions. However, no systematic mapping has been conducted in Germany to date. The aim of this study was therefore to systematically identify and analyze science–practice collaborations within the German PHS.

**Methodology:**

In a structured screening process, various data sources from 2015 to 2024 were systematically searched: (i) Google™, (ii) conference abstract volumes, (iii) scientific databases, (iv) databases for grey literature, supplemented by (v) an online survey, (vi) snowballing, and (vii) input from the “EvidenzOGD” project consortium network. Identified collaborations were categorized according to structural characteristics and analyzed descriptively using frequency analyses stratified by subgroups.

**Results:**

A total of 611 science–practice collaborations in the PHS were identified, most of them through conference contributions. The number of collaborations increased over the study period. Many focused on classical fields of PHS activities and were mainly implemented at the municipal level. Coordination was almost evenly distributed between academic institutions and PHS institutions, while implementation was predominantly located in western federal states.

**Conclusion:**

This mapping of science–practice collaborations provides a comprehensive overview of a complex and multifaceted landscape of collaboration. It offers a foundation for further in-depth analyses regarding facilitating and hindering factors of collaboration, for example, as well as a better understanding of thematic and geographical clustering.

## Einleitung

Die Verzahnung von Wissenschaft und Praxis gewinnt im Öffentlichen Gesundheitsdienst (ÖGD) zunehmend an Bedeutung. Als zentrale Instanz für Gesundheitsschutz und -förderung auf kommunaler, Landes- und Bundesebene spielt der ÖGD nicht nur in Gesundheitskrisen eine Schlüsselrolle. Eingebettet in die öffentliche Verwaltung kann er wesentlich zur Umsetzung evidenzbasierter Public-Health-Maßnahmen beitragen und evidenzinformierte Entscheidungsfindungsprozesse – beispielsweise im Rahmen datenbasierter Gesundheitsplanung – mitgestalten [1]. Kooperationen zwischen Wissenschaft und Praxis bieten hier erhebliches Potenzial, wissenschaftliche Erkenntnisse in konkrete Maßnahmen zu überführen und praxisrelevante Fragestellungen in der Forschung zu verankern.

Die Bedeutung solcher kooperativen Ansätze ist lange bekannt und wurde in den letzten Jahren auch politisch zunehmend anerkannt. Bereits 2016 machte der Bundesverband der Ärztinnen und Ärzte des Öffentlichen Gesundheitsdienstes (BVÖGD) auf den Investitionsbedarf zur Förderung von Wissenschaft-Praxis-Kooperationen aufmerksam und schlug einen modularen Ansatz zur Verzahnung von Praxis, Forschung und Lehre in der öffentlichen Gesundheit vor [2, 3]. Wenige Jahre darauf hob das Bundesministerium für Gesundheit (BMG) die Relevanz einer engen Verknüpfung von Forschung und Praxis im ÖGD hervor und definierte die aktive ÖGD-Beteiligung explizit als Förderkriterium in 2 Förderschwerpunkten [4, 5]. In seinem dritten Bericht zur strukturellen Weiterentwicklung des ÖGD verwies der Beirat zur Beratung zukunftsfähiger Strukturen im Öffentlichen Gesundheitsdienst in Umsetzung des Paktes für den Öffentlichen Gesundheitsdienst (Beirat Pakt ÖGD) zudem auf die Notwendigkeit, wissenschaftliche Kompetenzen und geeignete Infrastrukturen auch außerhalb projektbasierter Förderung vorzuhalten, um wissenschaftsbasierte Ansätze implementieren und Expertise auch kurzfristig verfügbar machen zu können [6]. Langfristige Kooperationen des ÖGD mit Hochschulen und außeruniversitären Forschungseinrichtungen wurden dabei als vielversprechender Ansatz hervorgehoben.

Trotz dieser Initiativen bleibt unklar, wie verbreitet solche Kooperationen im ÖGD sind, wie sie ausgestaltet werden und durch welche strukturellen Merkmale sie gekennzeichnet sind. Eine erste explorative Erhebung zu Wissenschaft-Praxis-Kooperationen im ÖGD wurde im Zukunftsforum Public Health (ZfPH) von 2018–2019 durchgeführt [7, 8]. Die Ergebnisse lieferten erste Einblicke in Kooperationsstrukturen, erlauben jedoch aufgrund der selektiven Dissemination innerhalb des Netzwerkes keine generalisierbaren Aussagen. Nach aktuellem Kenntnisstand existieren bislang keine systematischen Erhebungen zu bestehenden Kooperationen im ÖGD. Vorhandene Studien fokussieren meist auf Ansätze zur strukturellen Förderung der Zusammenarbeit oder den Aufbau fachlicher wie methodischer Kompetenzen [9–15]. Es fehlt eine umfassende Analyse, die aufzeigt, in welchen Bereichen, Formaten und Regionen kooperiert wird und welche Rolle ÖGD und Wissenschaft in gemeinsamen Forschungsansätzen einnehmen. Eine solche Analyse ist essenziell, um vorhandene Potenziale gezielter zu nutzen, effektive Fördermaßnahmen zu entwickeln und langfristig tragfähige Schnittstellen zwischen Wissenschaft und Praxis im ÖGD aufzubauen.

Vor diesem Hintergrund zielt die vorliegende Studie darauf ab, bestehende Kooperationen zwischen Wissenschaft und Praxis im ÖGD systematisch zu identifizieren, anhand ausgewählter Strukturmerkmale zu analysieren und Wissens- und Forschungslücken aufzudecken.

## Methodik

Zur Identifikation bestehender Kooperationen zwischen Wissenschaft und Praxis im ÖGD wurden von Juli 2021 bis März 2025 verschiedene Datenquellen systematisch gescreent, die im Zeitraum von 2015 bis 2024 veröffentlicht worden sind. Neben wissenschaftlichen wurden gezielt auch Quellen grauer Literatur durchsucht. Gemeint sind Veröffentlichungen außerhalb der klassischen akademischen Publikationskanäle, wie beispielsweise Konferenz‑, Tagungs- und Kongressbeiträge, Projekt- und Forschungsberichte, Webseiten, Diplom- und Masterarbeiten, Dissertationen oder auch Pressemitteilungen. Die Einschlusskriterien wurden wie folgt definiert: (1) Verfügbarkeit in Deutsch oder Englisch, (2) Durchführung der Kooperation im Zeitraum von 2015 bis 2024, (3) Beschreibung einer gemeinsamen, zweckgebundenen und zielgerichteten Bearbeitung von Aufgaben, Projekten, Prozessen oder wissenschaftlichen Fragestellungen und (4) Beteiligung von Akteur:innen sowohl aus der ÖGD-Praxis als auch aus der Wissenschaft. Als „ÖGD-Praxis“ wurden alle Akteursgruppen und Institutionen bezeichnet, die im deutschen Gesundheitswesen auf Landes- oder kommunaler Ebene mit einem spezifischen Mandat für Öffentliche Gesundheit, definiert als die öffentliche Sorge um die Gesundheit aller [16], ausgestattet sind. Da der Fokus auf Landes- und kommunaler Ebene lag, wurden ausschließlich Kooperationen eingeschlossen, bei denen untere (z. B. Gesundheitsämter) oder mittlere Gesundheitsbehörden (z. B. Landesgesundheitsämter oder -zentren) involviert waren. Kooperationen, an denen ÖGD-seitig ausschließlich Bundesinstitutionen, wie das Robert Koch-Institut (RKI) oder die Bundeszentrale für gesundheitliche Aufklärung (BZgA, jetzt Bundesinstitut für Öffentliche Gesundheit – BIÖG), beteiligt waren, wurden nicht systematisch miterfasst. Unter „Wissenschaft“ wurden sämtlich akademische Institutionen gefasst, die sich mit Fragen der öffentlichen Gesundheit befassen. Dazu zählten insbesondere universitäre und außeruniversitäre Forschungseinrichtungen und -institute.

**Abb. 1:**
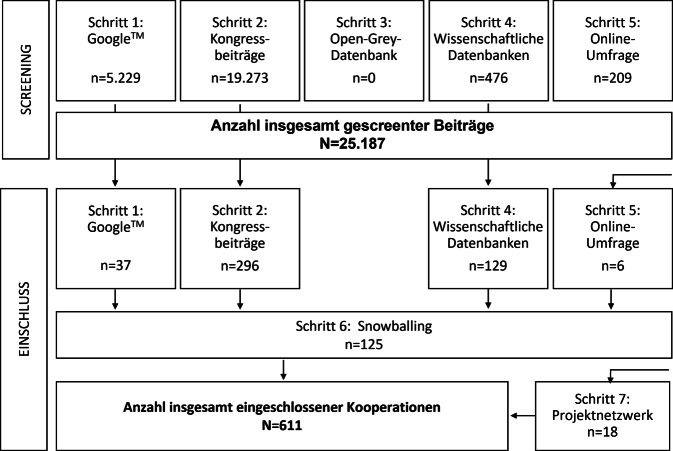
Screeningprozess zur Identifikation von Wissenschaft-Praxis-Kooperationen im Öffentlichen Gesundheitsdienst (ÖGD), Flowchart-Diagramm

Die Identifikation erfolgte in 7 teils parallel durchgeführten Schritten (Abb. 1). Die ersten 10 % der Suchtreffer in den Suchmaschinen und Datenbanken wurden immer nach dem 4‑Augen-Prinzip gescreent und Unstimmigkeiten wurden diskursiv geklärt. Bei ausreichender Übereinstimmung übernahm eine Person das weitere Screening und eine andere Person die Überprüfung.

Im *ersten Schritt* wurde eine systematische Suche in Google™ durchgeführt – zunächst für den Zeitraum 01/2015–08/2021 (Suche im August 2021), später erweitert auf 09/2021–11/2024 (Suche im November 2024). Hierzu wurden relevante Suchbegriffe vom Projektteam identifiziert, getestet, iterativ angepasst und anschließend in mehreren Suchblöcken miteinander kombiniert. Es wurde berücksichtigt, dass die maximale Anzahl der verwendbaren Suchbegriffe in Google™ auf 32 beschränkt ist. Für jeden Suchblock wurden die Anzahl gescreenter Beiträge, eingeschlossener Kooperationen sowie das Suchdatum dokumentiert (siehe zusätzliches Onlinematerial (ZOM) 1). Das Screening wurde bis zum Erscheinen des Hinweises zur Ergebnissättigung durchgeführt, der erscheint, sobald die weiteren Ergebnisse den bisherigen sehr ähnlich sind [17]. Zur Sicherstellung der Nachvollziehbarkeit und Reproduzierbarkeit wurden alle Einstellungen zur Personalisierung der Suche deaktiviert.

Im *zweiten Schritt* wurde ein Screening von Kongressbeiträgen durchgeführt. Grundlage bildeten die Abstract-Bände thematisch relevanter Kongresse, Tagungen und Symposien (Tab. 1), deren Titel und Abstracts systematisch gemäß oben definierter Einschlusskriterien gescreent wurden. Zur Überprüfung der Machbarkeit und zur Feinjustierung des Vorgehens wurde im Projektteam zunächst ein Probedurchlauf mit 2 Abstract-Bänden durchgeführt. Nach einer initialen Sichtung der Jahre 2015–2021 (Suche von 09/2021–05/2022) wurde das Screening schrittweise um jeweils ein weiteres Jahr bis 2024 erweitert.

**Tab. 1:** Kongresse, Tagungen und Symposien, die auf Wissenschaft-Praxis-Kooperationen gescreent wurden

Abkürzung	Kongresstitel	Ausrichter
AuG	Kongress Armut und Gesundheit	Gesundheit Berlin-Brandenburg e. V.
DGEpi	DGEpi-Jahrestagung	Deutsche Gesellschaft für Epidemiologie (DGEpi)
DGKH	Kongress für Krankenhaushygiene	Deutsche Gesellschaft für Allgemeine und Krankenhaushygiene (DGKH)
DGKJ	Kongress für Kinder- und Jugendmedizin	Deutsche Gesellschaft für Kinder- und Jugendmedizin (DGKJ), Deutsche Gesellschaft für Sozialpädiatrie und Jugendmedizin e. V. (DGSPJ), Berufsverband Kinderkrankenpflege Deutschland e. V. (BEKD), Deutsche Gesellschaft für Kinder- und Jugendchirurgie e. V. (DGKJCH), Gesellschaft für Pädiatrische Infektiologie e. V. (DGPI)
DGMS	Jahrestagung der Deutschen Gesellschaft für Medizinische Soziologie (DGMS) e. V.	Deutsche Gesellschaft für Medizinische Soziologie (DGMS) e. V.
DGSMP	Jahrestagung der Deutschen Gesellschaft für Sozialmedizin und Prävention (DGSMP) e. V.	Deutsche Gesellschaft für Sozialmedizin und Prävention (DGSMP) e. V.
DKVF	Deutscher Kongress für Versorgungsforschung	Deutsches Netzwerk für Versorgungsforschung (DNVF)
EbM	Jahrestagung des EbM-Netzwerks	Netzwerk Evidenzbasierte Medizin e. V. (EbM-Netzwerk)
GHUP	Jahrestagung der Gesellschaft für Hygiene, Umweltmedizin und Präventivmedizin (GHUP)	Gesellschaft für Hygiene, Umweltmedizin und Präventivmedizin (GHUP)
KIT	Kongress für Infektionskrankheiten und Tropenmedizin	Deutsche Gesellschaft für Infektiologie e. V., Deutsche Gesellschaft für Pädiatrische Infektiologie e. V., Deutsche Gesellschaft für Tropenmedizin, Reisemedizin und Globale Gesundheit e. V.
LGL	Bayerischer Kongress für den Öffentlichen Gesundheitsdienst	Bayerisches Landesamt für Gesundheit und Lebensmittelsicherheit (LGL)
ÖGD-Forum	Forum für den Öffentlichen Gesundheitsdienst	Robert Koch-Institut (RKI), Umweltbundesamt (UBA), Bundesinstitut für Risikobewertung (BfR)
ÖGD-Kongress	Wissenschaftlicher Kongress für den Öffentlichen Gesundheitsdienst	Bundesverband der Ärztinnen und Ärzte im Öffentlichen Gesundheitsdienst (BVÖGD), Bundesverbande der Zahnärztinnen und Zahnärzte im Öffentlichen Gesundheitsdienst (BZÖG), Deutsche Gesellschaft für Öffentliches Gesundheitswesen (DGÖG), Gesellschaft für Hygiene, Umweltmedizin und Präventivmedizin (GHUP)
PH3	Gemeinsame Tagung der Public-Health-Organisationen aus Österreich, Deutschland und der Schweiz	Österreichische Gesellschaft für Public Health, Deutsche Gesellschaft für Sozialmedizin und Prävention (DGSMP), Deutsche Gesellschaft für Public Health e. V. (DGPH), Swiss Public Health Doctors, Public Health Schweiz
Präventionskongress	Präventionskongress	Bundesministerium für Gesundheit (BMG), Bundesvereinigung Prävention und Gesundheitsförderung e. V. (BVPG)
ZfPH	Symposium des Zukunftsforums Public Health	Zukunftsforum Public Health (ZfPH)

Im *dritten Schritt* wurde eine Suche über die Online-Datenbank OpenGrey (System for Information on Grey Literature in Europe) durchgeführt. OpenGrey ermöglicht freien Zugriff auf Forschungsberichte und weitere graue Literatur und umfasst verschiedene Fachbereiche [18].

Im *vierten Schritt* wurde eine systematische Suche in den wissenschaftlichen Datenbanken PubMed und LIVIVO durchgeführt. Hierzu wurden relevante Suchbegriffe erarbeitet und in Suchblöcken, zunächst für 2015 bis 2022, später erweitert auf 2023 bis 2024, strukturiert (siehe ZOM 1). Die Treffer wurden anschließend mittels des webbasierten Screening-Tools „Rayyan“ [19] gescreent, wobei der erste Suchdurchlauf, der rund 80 % der Treffer umfasste, im 4‑Augen-Prinzip durchgeführt wurde.

Parallel erfolgte im *fünften Schritt* eine standardisierte Online-Befragung relevanter Akteur:innen via LimeSurvey, um auch nicht veröffentlichte Kooperationen zu identifizieren. Über den Survey bestand die Möglichkeit, freiwillig auf Kooperationen hinzuweisen, indem wenige Eckdaten – darunter Titel, Inhalte, beteiligte Institutionen, thematische Zuordnung, weiterführende Hinweise – übermittelt wurden. Die Dissemination erfolgte fortlaufend über verschiedene Veranstaltungen und Verteiler. Die Befragung begann im Juni 2022 und ist zum Zeitpunkt der Auswertung weiter offen [20]. Es wurden alle Kooperationen in die Auswertung aufgenommen, die bis 12/2024 eingegangen sind.

Im *sechsten Schritt* wurden mittels Snowballing alle eingeschlossenen Kooperationen gezielt auf Hinweise zu weiteren Kooperationen analysiert. Treffer wurden online recherchiert, hinsichtlich ihrer Relevanz verifiziert und gemäß den Einschlusskriterien aufgenommen.

Im *siebten Schritt* wurde implizites und informelles Wissen gezielt aus den beruflichen Netzwerken der Personen aus dem Projektkonsortium des EvidenzÖGD-Projektes [20] miteinbezogen.

### Analyse der Kooperationen.

Zur Analyse der identifizierten Kooperationen wurde ein Kategoriensystem mit zugehörigen Leitfragen konzipiert (Tab. 2) und durch einen Kodierleitfaden zur Erhöhung der internen Reliabilität ergänzt (siehe ZOM 2). Die Kategorien wurden zunächst deduktiv auf Basis von Brainstorming und ergänzender Literaturrecherche definiert und dann im Zuge der Kodierung induktiv weiterentwickelt. Die finale Kategorisierung erfolgte tabellarisch in Microsoft Excel. Jede Kodierung wurde von mindestens einer weiteren Person überprüft und etwaige Abweichungen wurden diskursiv gelöst. Im Anschluss wurden stratifizierte Häufigkeitsanalysen durchgeführt, bei denen die relativen und absoluten Verteilungen der Kooperationen entlang zentraler Strukturmerkmale ausgewertet wurden. Die Stratifikation erfolgte nach relevanten Vergleichsmerkmalen wie Art der Kooperation (z. B. unbefristet vs. projektbezogen), thematische Ausrichtung (z. B. Umweltgesundheit, Prävention, Infektionsschutz), Zeithorizont (z. B. laufend vs. abgeschlossen) sowie räumlicher Umsetzungsebene (kommunal, Landes- oder Bundesebene). Diese stratifizierten Analysen ermöglichen es, Verteilungsmuster und Schwerpunkte innerhalb der Kooperationslandschaft sichtbar zu machen und zugleich weniger erschlossene Themenbereiche zu identifizieren.

**Tab. 2:** Wissenschaft-Praxis-Kooperationen im Öffentlichen Gesundheitsdienst (ÖGD) – Merkmale und Anteile

Kategorie/Auswertungsparameter	Fragestellung	Unterkategorien	Anzahl	Anteil^a^^(%)^
**Kooperationsdauer**	*Über welche Dauer erstrecken sich wie viele Kooperationen?*	≤ 1 Jahr	28	13,3
		2–5 Jahre	112	53,3
		6–10 Jahre	42	20,0
		> 10 Jahre	28	13,3
		**Gesamt**^**d**^	**210**	**–**
		*k.* *A.*	*401*	–
**Kooperationsstatus**	*Welchem Status lassen sich wie viele Kooperationen zuordnen?*	Abgeschlossen	117	63,2
		Laufend	68	36,8
		**Gesamt**^**d**^	**185**	–
		*k.* *A.*	*426*	–
**Kooperationsform**	*Welche Kooperationsformen treten wie häufig auf?*	Veranstaltungsbezogene Kooperationen	160	26,2
		Publikationsbezogene Kooperationen	268	43,9
		Projektbezogene Kooperationen	126	20,6
		Unbefristete Kooperationen	57	9,3
		**Gesamt**^**d**^	**611**	–
**Publikationsform**	*Welche Formen der Veröffentlichung von Kooperationen treten wie häufig auf?*	Journal Article	161	27,6
		Vortrag	159	27,3
		Website	88	15,1
		Poster(‑Präsentation)	81	13,9
		Workshop	34	5,8
		Fachforum/Symposium	18	3,1
		Bericht	17	2,9
		Podiumsdiskussion	4	0,7
		Seminar	3	0,5
		Abschlussarbeit	3	0,5
		Stellungnahme	3	0,5
		Arbeitsgruppe	2	0,3
		Sonstiges	10	1,7
		**Gesamt**^**d**^	**583**	–
		*k.* *A.*	*28*	–
**Koordination/Erstautor:innenschaft**^**b**^	*Wie häufig liegt die Koordination/Erstautor:innenschaft der Kooperationen bei Personen mit welcher Affiliation?*	Wissenschaft	342	57,7
		ÖGD – kommunale Ebene	139	23,4
		ÖGD – Landesebene	117	19,7
		ÖGD – Bundesebene	37	6,2
		Sonstiges	8	1,3
		**Gesamt**^**d**^	**593**	–
		*k.* *A.*	*18*	–
**Co-Autor:innen**	*Wie viele weitere Personen sind an den Kooperationen beteiligt?*	Alleinige Autor:innenschaft	40	6,9
		1	53	9,2
		2	66	11,4
		3–5	201	34,8
		6–10	160	27,7
		≥ 10	57	9,9
		**Gesamt**^**d**^	**577**	–
		*k.* *A.*	*34*	–
**ÖGD-Ebene der Institutionen**^**c**^	*Wie häufig sind ÖGD-Institutionen von kommunaler, Landes- oder Bundesebene an Kooperationen beteiligt?*	Kommunale Ebene	439	73,3
		Landesebene	256	42,7
		Bundesebene	72	12,0
		**Gesamt**^**d**^	**599**	–
		*k.* *A.*	*12*	–
**Umsetzungsebene**^**c**^	*Wie häufig findet die Umsetzung der Kooperationen auf welcher *Verwaltungsebene *statt?*	Kommunale Ebene	355	68,7
		Landesebene	127	24,6
		Bundesebene	85	16,4
		**Gesamt**^**d**^	**517**	–
		*k.* *A.*	*94*	–
**Bundesländer**^**c**^	*In welchem Bundesland werden wie häufig Kooperationen umgesetzt?*	Nordrhein-Westfalen	97	24,9
		Baden-Württemberg	88	22,6
		Bayern	82	21,0
		Hessen	57	14,6
		Niedersachsen	36	9,2
		Berlin	33	8,5
		Hamburg	23	5,9
		Bremen	15	3,8
		Sachsen	15	3,8
		Rheinland-Pfalz	13	3,3
		Schleswig-Holstein	13	3,3
		Brandenburg	7	1,8
		Mecklenburg-Vorpommern	6	1,5
		Sachsen-Anhalt	5	1,3
		Saarland	4	1,0
		Thüringen	4	1,0
		**Gesamt**^**d**^	**390**	–
		*k.* *A.*	*221*	–
**Kooperierende Institutionen**^**c**^	*Wie häufig sind Institutionen welcher Institutionsformen in Kooperationen involviert gewesen?*	Universitäten, Hochschulen, Akademien, Institute	1039	33,0^e^
		Kommunalverwaltung	837	26,6^e^
		Landesverwaltung	301	9,6^e^
		Gesundheitseinrichtungen	252	8,0^e^
		Nichtuniversitäre Forschungseinrichtungen/-institute	246	7,8^e^
		Fachgesellschaften und Netzwerke	95	3,0^e^
		Berufsverbände und -vertretungen	44	1,4^e^
		Krankenkassen	41	1,3^e^
		Bundesverwaltung	38	1,2^e^
		Wohlfahrtsverbände und Stiftungen	35	1,1^e^
		Privatwirtschaftliche Institutionen	19	0,6^e^
		Sonstige Institutionen	202	6,4^e^
		**Gesamt**	**3149**	–

^a^ Bezieht sich auf die gültigen Kooperationen in der jeweiligen Kategorie, wobei die K.-A.-Angaben rausgerechnet werden

^b^ Mehrfachaffiliationen sind möglich, wenn Personen gleichzeitig für Institutionen aus Praxis und Wissenschaft tätig sind

^*c*^ Mehrfachangaben möglich

^d^ Die Gesamtzahl schließt alle gültigen Fälle ein (= Gesamtzahl der Kooperationen abzüglich der K.-A.-Angaben)

^e^ Die prozentualen Angaben beziehen sich auf den Anteil der jeweiligen Institutionsform an der Gesamtzahl der erfassten institutionellen Beteiligungen (*N* = 3149)

*k.* *A.* keine Angabe

### Qualitätssicherung.

Zur Sicherstellung von Intersubjektivität wurde ein reflexiver, interdisziplinärer Ansatz gewählt. Ein Review-Protokoll wurde nicht vorab registriert, da es sich nicht um eine systematische Übersichtsarbeit im engeren Sinne handelt. Ziele waren die umfassende Identifikation, Kartierung und Beschreibung von Wissenschaft-Praxis-Kooperationen im ÖGD auf Grundlage ausschließlich öffentlich zugänglicher Informationen. Entsprechend wurden keine personenbezogenen Daten erhoben. Die Beschreibung der Inhalte dieser Studie erfolgte in Anlehnung an die Checkliste Preferred Reporting Items for Systematic Reviews and Meta-Analyses Extension for Scoping Reviews (PRISMA-ScR; [21]), welche sich ausgefüllt im Anhang befindet (siehe ZOM 3).

## Ergebnisse

Insgesamt wurden *25.187* Einzelbeiträge gescreent. Den größten Anteil machte dabei das Kongressscreening *(n* *=* *19.273)* aus, gefolgt von dem Google™-Screening (*n* = 5229), dem Screening in den wissenschaftlichen Datenbanken *(n* *=* *476)* und dem Online-Survey *(n* *=* *209)*. Trotz mehrerer Durchläufe mit zentralen Suchbegriffen (z. B. ÖGD, Gesundheitsamt, Gesundheitsdienst) konnten über die Online-Datenbank OpenGrey keine relevanten Treffer erzielt werden, weshalb dieser Schritt nicht weiterverfolgt wurde. Insgesamt wurden *611* Kooperationen eingeschlossen, die meisten mittels Kongressscreenings *(n* *=* *296)*, gefolgt von dem Screening wissenschaftlicher Datenbanken *(n* *=* *129)* und dem Snowballing *(n* *=* *125).* Über das Google™-Screening *(n* *=* *37)*, das Projektnetzwerk *(n* *=* *18)* und den Online-Survey *(n* *=* *6)* wurden deutlich weniger Kooperationen identifiziert (Abb. 1).

Eine Übersicht der Ergebnisse hinsichtlich der zentralen Kategorien bzw. Auswertungsparameter ist in Tab. 2 zu finden. Eine detaillierte Darstellung befindet sich in ZOM 4. Die prozentualen Angaben beziehen sich dabei immer auf die Gesamtzahl der gültigen Kooperationen (= Gesamtzahl der Kooperationen abzüglich der *K.-A*.-Fälle).

### Zeitverlauf, Kooperationsdauer und -status.

**Abb. 2:**
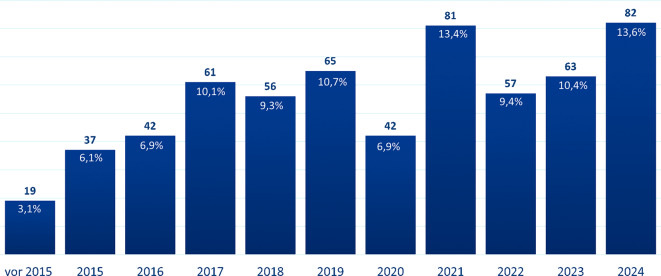
Absolute und relative Anzahl identifizierter Wissenschaft-Praxis-Kooperationen in den Jahren 2015–2024. *Hinweis*: Kooperationen, die vor 2015 begonnen wurden, im Erhebungszeitraum jedoch noch aktiv waren, sind in der Abbildung nicht enthalten (*n* = 19). Ebenso nicht enthalten sind Kooperationen, bei denen der genaue Beginn nicht identifiziert werden konnte (*n* *=* 6)

Von 2015 bis 2024 zeigt sich in der Tendenz eine Zunahme an Kooperationen (Abb. 2). Die kooperationsstärksten Jahre sind dabei 2021 *(n* *=* *81)* und 2024 *(n* *=* *82)*. Es wurden einige Kooperationen identifiziert, die außerhalb des Suchzeitraums starteten, sich aber bis in den Zeitraum der Suche hinein erstrecken *(n* *=* *19)*. Für Kooperationen mit ermittelbarer Dauer lag die durchschnittliche Laufzeit bei 5,8 Jahren. Am häufigsten waren Kooperationen mit einer Dauer von 2–5 Jahren *(n* *=* *112), *während langfristige Kooperationen mit einer Laufzeit von über 10 Jahren selten waren *(n* *=* *28)*. Für den Großteil konnte die Dauer nicht identifiziert werden *(n* *=* *401)*. Zum Analysezeitpunkt waren *117* Kooperationen abgeschlossen, *68* liefen noch, bei *426 *lagen keine Angaben zum Status vor.

### Kooperations- und Publikationsform.

Am häufigsten erfolgte die Zusammenarbeit in publikationsbezogenen Kooperationen *(n* *=* *268)*, definiert als zeitlich begrenzte Zusammenarbeit, deren Ergebnisse gemeinsam veröffentlicht werden. Auch veranstaltungsbezogene Kooperationen *(n* *=* *160)*, die eine einmalige, zeitlich begrenzte Zusammenarbeit zur Planung und Durchführung einer spezifischen Veranstaltung (z. B. Vortrag, Workshop) beinhalten, waren häufig. Zeitlich begrenzte, zielgerichtete Kooperationen im Rahmen eines Projekts, die mit dem Abschluss des Projektzeitraums oder nach Erreichen der Meilensteine enden, machten rund ein Fünftel der identifizierten Beiträge aus *(n* *=* *126)*. Deutlich seltener waren zeitlich unbefristete Kooperationen *(n* *=* *57)*, die sich durch eine langfristige Zusammenarbeit ohne festes Enddatum auszeichnen und häufig nur zu bestimmten Anlässen oder in bestimmten Zyklen aktiv werden (z. B. Netzwerke, Forschungsverbünde).

Kooperationen wurden überwiegend in Form von wissenschaftlichen Artikeln in Fachzeitschriften *(n* *=* *161)* oder über Vorträge *(n* *=* *159)*, z. B. auf Fachkongressen, sichtbar. Weitere Publikationsformen waren Webseiten *(n* *=* *88)* und Posterpräsentationen *(n* *=* *81)*.

### Rollenverteilung in Kooperationen.

Die Autor:innenschaft kann ein erster Anhaltspunkt für die Rollenverteilung innerhalb von Kooperationen sein. Bei Einzelbeiträgen wurde die Erstautor:innenschaft betrachtet, bei Projekt- und Netzwerkkooperationen die Affiliation der Projekt- oder Netzwerkleitung. Bei etwas mehr als der Hälfte der Kooperationen lag die Erstautor:innenschaft bzw. Koordination bei Personen mit wissenschaftlicher Affiliation (z. B. Universität, Hochschule; *n* *=* *342)* und bei etwas weniger als der Hälfte der Kooperationen *(n* *=* *293) *bei Personen aus dem ÖGD*,* wobei die kommunale Ebene am stärksten vertreten war (*n* *=* *119*). Überschneidungen ergaben sich durch Mehrfachaffiliationen einzelner Personen (z. B. Gesundheitsamt und Hochschule). Im Durchschnitt waren etwa 6 Co-Autor:innen bzw. weitere Personen über die koordinierende Person oder Erstautor:in hinaus an der Kooperation beteiligt. Am häufigsten lag dieser Anteil bei 3–5 Personen *(n* *=* *201)*. Nur in wenigen Kooperationen waren 10 oder mehr Personen *(n* *=* *57)* zusätzlich beteiligt. In einem kleinen Anteil war keine weitere Person *(n* *=* *40)* beteiligt, was sich in allen Fällen durch Mehrfachaffiliationen begründen lässt.

### ÖGD-Ebene der Institutionen und Umsetzungsebene.

Ein Großteil der Kooperationen erfolgte unter Beteiligung des kommunalen ÖGD *(n* *=* *439)*, gefolgt von der Landesebene *(n* *=* *256). *Bei *72* Kooperationen war zusätzlich auch noch die Bundesebene beteiligt. Da einzelne Kooperationen häufig Institutionen mehrerer Verwaltungsebenen umfassten – etwa in Fällen, in denen sowohl ein kommunales Gesundheitsamt als auch ein Landesgesundheitsamt beteiligt waren –, wurden diese mehrfach gezählt.

Auch hinsichtlich der Umsetzung fanden die meisten Kooperationen auf kommunaler Ebene statt *(n* *=* *355)*, gefolgt von der Landesebene *(n* *=* *127)* und der Bundesebene *(n* *=* *85)*. In vielen Fällen fand die Umsetzung übergreifend auf mehreren Verwaltungsebenen statt, wenn z. B. Daten auf kommunaler Ebene erfasst und auf Landesebene gebündelt werden, um im Anschluss in Zusammenarbeit mit einer Universität weitere Auswertungen zu initiieren.

### Thematische Einordnung.

**Abb. 3:**
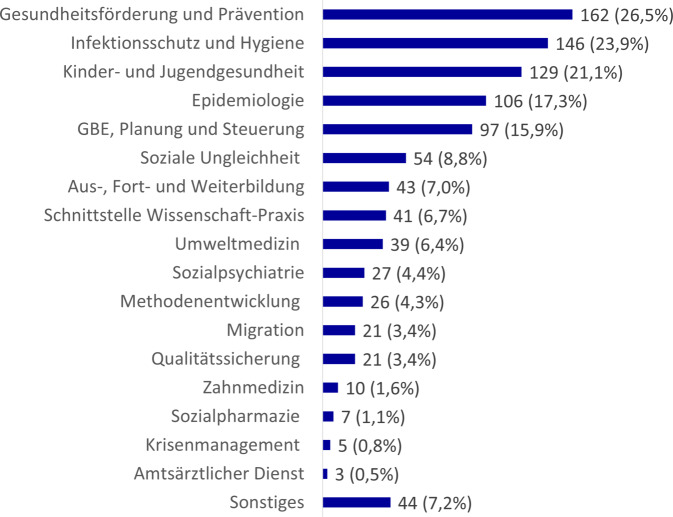
Thematische Einordnung der Kooperationen zwischen Wissenschaft und Praxis. (Zusätzlich zur absoluten Anzahl ist prozentual der Anteil an der Gesamtzahl an Kooperationen (*N* = 611) dargestellt, da Mehrfachnennungen möglich waren.) *GBE* Gesundheitsberichterstattung

Die thematische Auswertung zeigt eine deutliche Schwerpunktsetzung in klassischen Handlungsfeldern des ÖGD (Abb. 3). Am häufigsten waren Kooperationen in den Bereichen Gesundheitsförderung und Prävention *(n* *=* *162)*, Infektionsschutz und Hygiene *(n* *=* *146) *sowie Kinder- und Jugendgesundheit *(n* *=* *129)*. Auch Epidemiologie *(n* *=* *106)* und Gesundheitsberichterstattung, Planung und Steuerung *(n* *=* *97) *bildeten häufig inhaltliche Schwerpunkte. Darüber hinaus zeigt sich ein relevanter Anteil an Kooperationen in strukturellen Querschnittsthemen. Dazu zählen Vorhaben, die sich inhaltlich mit Strukturen an der Schnittstelle von Wissenschaft und Praxis auseinandersetzen *(n* *=* *41)*, solche in der Aus‑, Fort- und Weiterbildung *(n* *=* *43)*, der Methodenentwicklung *(n* *=* *26)* sowie der Qualitätssicherung *(n* *=* *21)*. Weniger häufig waren Kooperationen zu den ÖGD-Themenbereichen Zahnmedizin *(n* *=* *10)*, Sozialpharmazie *(n* *=* *7)* oder amtsärztlicher Dienst *(n* *=* *3).* Da Kooperationen meistens mehrere Themenbereiche adressierten, kam es oft zu Mehrfachzuordnungen.

### Bundesländer.

Die geografische Verortung der Umsetzungen der Kooperationen konzentrierte sich auf die Flächenländer Nordrhein-Westfalen *(n* *=* *97),* Baden-Württemberg *(n* *=* *88)* und Bayern *(n* *=* *82),* gefolgt von Hessen *(n* *=* *57)*, Niedersachsen *(n* *=* *36)* und Berlin *(n* *=* *33; *Tab. 2). In den anderen Bundesländern gab es nur sehr wenige Kooperationen. Es ist zu beachten, dass für *221 *Kooperationen keine eindeutige geografische Verortung vorgenommen werden konnte.

### Kooperierende Institutionen.

In den 611 Kooperationen wurden insgesamt 3149 institutionelle Beteiligungen gezählt. Dabei wurde jede Institution für jede Kooperation, an der sie beteiligt war, separat erfasst. Mehrfachbeteiligungen derselben Institution wurden dementsprechend mehrfach berücksichtigt. Jede Institution wurde einer Institutionsform zugeordnet. Am häufigsten vertreten waren wissenschaftliche Einrichtungen wie Universitäten, Hochschulen, Akademien und Institute *(n* *=* *1039)*, gefolgt von Kommunalverwaltungen *(n* *=* *837)* und Landesverwaltungen *(n* *=* *301)*. Auch Gesundheitseinrichtungen *(n* *=* *252) *sowie nichtuniversitäre Forschungseinrichtungen und -institute *(n* *=* *246) *waren häufig vertreten. Andere Institutionsformen, wie z. B. Krankenkassen oder Berufsverbände, waren deutlich seltener an Kooperationen beteiligt.

## Diskussion

Mithilfe des mehrstufigen methodischen Vorgehens konnten im Zeitraum von 2015 bis 2024 insgesamt 611 Kooperationen identifiziert werden, die zwischen Wissenschaft und Praxis initiiert bzw. durchgeführt wurden. Besonders ergiebig war dabei das Screening von Kongressbeiträgen und wissenschaftlichen Datenbanken.

Die Analyse zeigt eine tendenziell zunehmende Anzahl von Kooperationen über den betrachteten Zeitraum, mit Spitzen in den Jahren 2021 und 2024. Der Rückgang in 2020 ist wahrscheinlich vorrangig dadurch zu erklären, dass bedingt durch die COVID-19-Pandemie nur wenige Kongresse stattfanden, deren Abstracts die Datengrundlage dieser Studie sind. Neben pandemiebedingten Impulsen dürften auch laufende Reform- und Modernisierungsprozesse im ÖGD zu dieser Dynamik beigetragen haben [22, 23]. Gleichzeitig könnte der Anstieg als erster Indikator für ein gewachsenes gegenseitiges Interesse an wissenschaftsbasierter Praxis und praxisorientierter Forschung gewertet werden.

Die Zusammenarbeit erfolgte überwiegend im Rahmen von zeitlich befristeten, publikations- oder veranstaltungsbezogenen Formaten. Solche projekt- bzw. anlassbezogenen Kooperationen zielen möglicherweise eher auf eine kurzfristige Generierung und Verbreitung von Wissen ab. Gleichzeitig zeigt die vergleichsweise geringe Anzahl an zeitlich unbegrenzten Kooperationen, dass längerfristige und strukturell verankerte Kooperationen, etwa im Rahmen von Netzwerken oder thematischen Arbeitsgruppen, noch ausgebaut werden können. Für eine nachhaltige Verankerung von Wissenschaft-Praxis-Kooperationen wären spezifische Förderformate wünschenswert, die nicht nur Einzelprojekte, sondern auch dauerhafte Strukturen wie Netzwerke, gemeinsame Personalstellen sowie (über-)regionale Forschungsverbünde ermöglichen [6, 13, 24].

Die regionale Analyse zeigt eine Häufung von Kooperationen in bevölkerungsreichen westdeutschen Flächenländern wie Nordrhein-Westfalen, Baden-Württemberg und Bayern. Mögliche Gründe hierfür könnten in der dort höheren Dichte wissenschaftlicher und behördlicher Institutionen [25, 26] sowie größeren Personalressourcen [27] liegen. Dabei ist zu berücksichtigen, dass sich die zugrunde liegenden Quellen auf absolute Zahlen beziehen und somit keine Aussagen über die relative Kooperationsintensität – etwa im Verhältnis zur Anzahl von Gesundheitsämtern oder Hochschulen pro Bundesland – zulassen. Dennoch könnte die vergleichsweise geringe Zahl an identifizierten Kooperationen in ostdeutschen Bundesländern auf bislang ungenutzte Potenziale hinweisen. Diese ließen sich möglicherweise durch gezielte Maßnahmen zur Stärkung kooperativer Strukturen innerhalb, zwischen und über Regionen hinweg erschließen. Zu beachten ist hierbei aber, dass für mehr als ein Drittel der Kooperationen keine gesicherte geografische Verortung vorgenommen werden konnte.

Die thematische Analyse zeigt Schwerpunkte in klassischen ÖGD-Aufgabenfeldern, insbesondere Gesundheitsförderung und Prävention, Infektionsschutz und Hygiene sowie Kinder- und Jugendgesundheit sind stark vertreten. Vergangene Erhebungen zeigten ähnliche Tendenzen [7, 8]. Die hohe Zahl an Kooperationen im Bereich des Infektionsschutzes könnte unter anderem im Kontext der COVID-19-Pandemie zu interpretieren sein. Diese hat die Bedeutung von Kooperationen zur Bewältigung gesundheitlicher Krisen und der Stärkung des ÖGD sowie den Bedarf an wissenschaftsbasierter Praxis verstärkt, was sich in einer Vielzahl an Förderausschreibungen widerspiegelt [4, 5, 28–30]. Die Notwendigkeit eines wissenschaftsbasierten und kooperationsstarken ÖGD, der als gestaltender Akteur vor Ort agieren kann, beschränkt sich aber natürlich nicht auf COVID-19 oder den Infektionsschutz, was sich durch die Themenvielfalt der Kooperationen zeigt, aber auch bereits wiederholt in Politik und Fachöffentlichkeit gefordert wurde [2, 3, 5, 15]. Auch das von der Gesundheitsministerkonferenz bereits 2018 verabschiedete Leitbild „Der ÖGD – Public Health vor Ort“ skizziert einen modernen ÖGD, der neben klassischem Gesundheitsschutz verstärkt beratende, bevölkerungsmedizinische und präventive Aufgaben sowie die Vernetzung lokaler Akteur:innen übernimmt [31]. Hinzu kommt, dass gerade in den kooperationsstarken Arbeitsbereichen ein großer Stellenaufwuchs im Rahmen des Förderprogramms „ÖGD-Pakt“ stattgefunden hat – auch hier verstärkt in westdeutschen Flächenländern. Das zeigt die Verteilung der aus Paktmitteln geschaffenen und besetzten Stellen nach Aufgabenbereichen bis Ende 2023 [32, 33]. Hier wird es von zentraler Bedeutung sein, dass mit Auslaufen des Paktes Ende 2026 sichergestellt wird, dass der Personalaufwuchs bzw. -erhalt auch darüber hinaus gesichert ist.

Die Ergebnisse zeigen auch, dass Querschnittsthemen wie Methodenentwicklung, Qualifikation oder Qualitätssicherung, die viele der klassischen Aufgabenfelder durchdringen, seltener als eigenständige Schwerpunkte in Kooperation gesetzt wurden. Dies deutet auf ein weiteres nicht ausgeschöpftes Forschungs- und Entwicklungspotenzial hin. Der Beirat für den ÖGD-Pakt empfiehlt in diesem Zusammenhang eine stärkere Förderung der Entwicklung evidenzbasierter fachlicher Standards und Qualitätssicherungsmaßnahmen für alle zentralen Aufgabenfelder des ÖGD [6], was sich in aktuellen Debatten zu Leitlinien im ÖGD widerspiegelt [29, 34]. Im Bereich Aus‑, Fort- und Weiterbildung sind in den vergangenen Jahren viele Forschungsvorhaben initiiert und durchgeführt worden [35–40], die weiter gestärkt und ausgebaut werden sollten – insbesondere auch vor dem Hintergrund der laufenden Professionalisierungs- und Qualifikationsdebatten im ÖGD.

Auch wenn die explizite Rolle des ÖGD innerhalb der Kooperationen im Rahmen dieser Erhebung nicht systematisch erfasst wurde, erlaubt die Analyse der beteiligten Institutionen oder der Affiliation der koordinierenden Person erste Rückschlüsse zur Rollenverteilung. Die Analyse zeigt, dass es in der Verteilung der Erstautor:innenschaft keine großen Unterschiede gibt. Perspektivisch erscheint es sinnvoll, Wege des Wissenstransfers innerhalb der Kooperationen differenzierter zu untersuchen – etwa im Hinblick darauf, ob ÖGD-Institutionen primär als Forschungsgegenstand (Forschung *über* den ÖGD) oder aktiv als Praxispartner (Forschung *mit* oder *durch* den ÖGD) beteiligt sind.

### Stärken und Limitationen

Dies ist nach unserem Kenntnisstand die erste systematische Erhebung von Kooperationen zwischen Wissenschaft und Praxis im ÖGD über einen längeren Zeitraum. Durch die mehrschrittige Herangehensweise konnte eine Vielzahl an Kooperationen identifiziert und auf Basis ausgewählter Strukturmerkmale analysiert werden. Insbesondere der Einbezug grauer Literatur erwies sich als zentral, da so auch Kooperationen abgebildet wurden, die in wissenschaftlichen Datenbanken nicht erfasst sind.

Dennoch sind einige Limitationen zu beachten. Kooperationen, über die bisher nicht öffentlich berichtet wurde, wurden nur dann eingeschlossen, wenn sie über das Projektnetzwerk oder die Online-Umfrage identifiziert wurden. Daraus ergibt sich ein potenzieller Selektionsbias, insbesondere wenn bestimmte Kooperationen über bisher noch nicht berücksichtigte Fachkongresse kommuniziert wurden. Es ist anzunehmen, dass die Online-Erhebung diesen Effekt teilweise kompensiert hat. Es ist angedacht, weitere themenspezifische Kongresse in das Screening einzubeziehen, um eine umfassende Erhebung zu gewährleisten.

Es lagen dem Forschungsteam für 7 Kongresse (von insgesamt 97) auch nach Rücksprache mit den Veranstalter:innen keine vollständigen Abstracts vor. Da bei diesen Kongressen jedoch in anderen Jahren nur wenige einschlägige Beiträge identifiziert wurden, wird der Einfluss auf die Gesamtergebnisse als gering bewertet.

Des Weiteren beschränkte sich das Screening in wissenschaftlichen Datenbanken auf PubMed und LIVIVO, wodurch Veröffentlichungen in anderen Datenbanken nicht identifiziert werden konnten. Mit der mehrschrittigen Suchstrategie wurde dem jedoch entgegengewirkt.

Ein weiterer potenziell limitierender Aspekt betrifft die Datenqualität in den Auswertungskategorien Kooperationsstatus, Kooperationsdauer und Bundesland, denn hier lagen häufig keine Angaben vor. Für alle anderen Parameter fiel der Anteil an Kooperationen, die sich nicht zuordnen ließen, deutlich geringer und somit vollständiger aus.

Da im zeitlichen Verlauf mehrere Personen in das Screening involviert waren (*n* = 8), bestand potenziell das Risiko für abweichende Zuordnungen. Dieses wurde durch die frühzeitige Definition klarer Ein- und Ausschlusskriterien, die Entwicklung eines einheitlichen Kodierleitfadens (siehe ZOM 2) sowie regelmäßige interne Absprachen minimiert.

## Fazit

Die vorliegende Analyse bietet erstmals eine systematische Erhebung von Kooperationen zwischen Wissenschaft und Praxis im ÖGD in Deutschland. Sie ermöglicht fundierte Einblicke in Struktur, Thematik, institutionelle Beteiligung und (bedingt) die regionale Verteilung der Kooperationslandschaft im ÖGD. Durch die Berücksichtigung ausgewählter Strukturmerkmale wird ein bislang nur punktuell sichtbares Feld in seiner Gesamtheit besser erfassbar. Die Ergebnisse zeigen die Vielfalt bestehender Kooperationen, machen aber auch Entwicklungspotenziale sichtbar – insbesondere im Hinblick auf längerfristige, strukturell verankerte Formate sowie die stärkere Einbindung strukturbezogener Querschnittsthemen.

Aufbauend auf den Erkenntnissen bietet sich Potenzial für vertiefende Analysen – etwa zu den Rollenverteilungen zwischen Wissenschaft und Praxis oder zur Identifikation von thematischen Forschungslücken. Aufbauend auf den Ergebnissen ist perspektivisch die Entwicklung eines systematischen Monitorings und einer Kooperationsdatenbank vorgesehen. Langfristig könnte die Verstetigung dieses Kooperationsscreenings dazu beitragen, Transparenz über bestehende Aktivitäten zu erhöhen, Akteur:innen miteinander zu vernetzen, strategische Steuerung im Kooperationsgeschehen des ÖGD zu unterstützen sowie Synergie- und Lerneffekte zu initiieren, um so langfristig den Wissenstransfer im ÖGD zu fördern.

## Supplementary Information

ESM 1: Suchbegriffe und -kombinationen in der systematischen Suche in wissenschaftlichen Datenbanken und über GoogleTM; ESM 2: Ausführliches Kodierschema zur Analyse der Kooperationen zwischen Wissenschaft und Praxis im ÖGD; ESM 3: Ausgefüllte PRISMA-ScR-Guideline(Preferred Reporting Items for Systematic reviews and Meta-Analyses extension for Scoping Reviews)-Checkliste; ESM 4: Ausführliche Ergebnistabelle des Kooperationsscreenings

## Data Availability

Alle Daten, die erforderlich sind, um die berichteten Ergebnisse zu interpretieren, sind im Manuskript enthalten.

## References

[1] Arnold L, Starke D (2021) Evidenzinformiertes Planen für Gesundheit – Koordination und Steuerung. In: Klapper B, Cichon I (Hrsg) Neustart! Für die Zukunft unseres Gesundheitswesens. MWV, Berlin, S 581–588

[2] Teichert U, Kaufhold C, Rissland J, Tinnemann P, Wildner M (2016) Vorschlag für ein bundesweites Johann-Peter Frank Kooperationsmodell im Rahmen der nationalen Leopoldina-Initiative für Public Health und Global Health. Gesundheitswesen 78(7):473–476. 10.1055/s-0042-109162 27438163 10.1055/s-0042-109162

[3] Bundesverband der Ärztinnen und Ärzte des Öffentlichen Gesundheitsdienstes e. V. (BVÖGD) (2021) Vorschlag Johann-Peter Frank Kooperationsmodell. https://www.bvoegd.de/jpf-kooperationsmodell/. Zugegriffen: 16. Juli 2021

[4] Bundesministerium für Gesundheit (BMG) (2022) Strukturelle Stärkung und Weiterentwicklung des Öffentlichen Gesundheitsdienstes (ÖGD). Öffentliche Förderbekanntmachung des Bundesministeriums für Gesundheit (BMG) veröffentlicht am 10.08.2022. https://projekttraeger.dlr.de/de/foerderung/foerderangebote-und-programme/staerkung-der-zusammenarbeit-zwischen-oeffentlichem-gesundheitsdienst-und-public-health. Zugegriffen: 22. Jan. 2024

[5] Bundesministerium für Gesundheit (BMG) (2020) Stärkung der Zusammenarbeit zwischen Öffentlichem Gesundheitsdienst und Public-Health-Forschung. Öffentliche Bekanntmachung des Bundesministeriums für Gesundheit (BMG) veröffentlicht am 27.02.2020. https://projekttraeger.dlr.de/de/foerderung/foerderangebote-und-programme/staerkung-der-zusammenarbeit-zwischen-oeffentlichem-gesundheitsdienst-und-public-health. Zugegriffen: 22. Jan. 2024

[6] Beirat Pakt ÖGD (2023) Wissenschaft und Forschung im und für einen zukunftsfähigen ÖGD. Berlin. Strukturelle und zukunftsorientierte Weiterentwicklung des Öffentlichen Gesundheitsdienstes. https://www.bundesgesundheitsministerium.de/themen/gesundheitswesen/pakt-fuer-den-oegd/beirat-pakt-oegd. Zugegriffen: 22. Jan. 2024

[7] Zukunftsforum Public Health (2025) Befragung: Kooperation zwischen Wissenschaft und Praxis im ÖGD – Zukunftsforum Public Health. https://zukunftsforum-public-health.de/befragung-kooperation-zwischen-wissenschaft-und-praxis-im-oegd/. Zugegriffen: 11. Juni 2025

[8] Zukunftsforum Public Health (2025) Plenarpräsentationen des 3. Symposiums des Zukunftsforums Public Health – Zukunftsforum Public Health. https://zukunftsforum-public-health.de/symposien/symposium-2019/dokumente/. Zugegriffen: 11. Juni 2025

[9] Bundesministerium für Gesundheit (BMG) (2025) Zusammenarbeit im ÖGD BW für mehr Synergie und Qualität (ZUSYNQ) | BMG. https://www.bundesgesundheitsministerium.de/ministerium/ressortforschung/handlungsfelder/forschungsschwerpunkte/strukturelle-staerkung-oegd/zusynq.html. Zugegriffen: 11. Juni 2025

[10] Schütz N, Schade M (2025) Kooperationen zwischen Hochschulen und dem Öffentlichen Gesundheitsdienst im Rahmen der Gesundheitsberichterstattung: Mehrwert für beide Kooperationspartner? Public Health Forum 33(1):60–63. 10.1515/pubhef-2024-0152

[11] Piontkowski E, Richter H, Bischof J, Herrmann A, Preiser C, Häske D et al (2024) Versorgungsforschung im Gesundheitsamt – eine explorative Interviewstudie zur wissenschaftlichen Methodenkompetenz im ÖGD in Baden-Württemberg. Gesundheitswesen. 10.1055/a-2308-7059 38631383 10.1055/a-2308-7059 PMC11404344

[12] OEGDforte (2025) OEGD-FORTE – Forschungs‑, Trainings- und Evidenznetzwerk für die öffentliche Gesundheit. https://oegd-forte.de/. Zugegriffen: 11. Juni 2025

[13] Arnold L, Bimczok S, Clemens T, Brand H, Starke D (2024) Implementing evidence ecosystems in the public health service: Development of a framework for designing tailored training programs. PLoS ONE 19(4):e292192. 10.1371/journal.pone.0292192 38635845 10.1371/journal.pone.0292192 PMC11025971

[14] Bundesministerium für Gesundheit (BMG) (2025) Nachhaltige Weiterentwicklung von Kompetenzen und Methoden am Beispiel SOPESS als Teil der Schuleingangsuntersuchung (KOMET-SEU) | BMG. https://www.bundesgesundheitsministerium.de/ministerium/ressortforschung/handlungsfelder/forschungsschwerpunkte/oegd-public-health-forsch/komet-seu.html. Zugegriffen: 11. Juni 2025 10.1055/a-2577-9620 40355107

[15] Bruns-Philipps E, Pohlabeln H, Hoopmann M, Reinke F, Windorfer A (2005) Der öffentliche Gesundheitsdienst als Kooperationspartner in der Prävention. Bundesgesundheitsblatt Gesundheitsforschung Gesundheitsschutz 48(10):1153–1161. 10.1007/s00103-005-1138-y 16172786 10.1007/s00103-005-1138-y

[16] Brand A, Stöckel S (2002) Die öffentliche Sorge um die Gesundheit aller – ein sinnvoller Anspruch? In: Brand A, von Engelhardt D, Simon A, Wehkamo KH (Hrsg) Individuelle Gesundheit versus public health?: Jahrestagung der Akademie für Ethik in der Medizin e. V., Hamburg 2001. Lit, Münster, S 11–28

[17] Erxleben C (2019) 5 Milliarden Suchergebnisse bei Google? Von wegen! BASIC thinking. https://www.basicthinking.de/blog/2019/03/08/google-suchergebnisse/. Zugegriffen: 11. Juni 2025

[18] OPENGREY (2025) OPENGREY.EU – Grey Literature Database. https://opengrey.eu/. Zugegriffen: 11. Juni 2025

[19] Ouzzani M, Hammady H, Fedorowicz Z, Elmagarmid A (2016) Rayyan‑a web and mobile app for systematic reviews. Syst Rev. 10.1186/s13643-016-0384-4 27919275 10.1186/s13643-016-0384-4 PMC5139140

[20] Akademie für Öffentliches Gesundheitswesen (AÖGW) (2023) EvidenzÖGD: Forschungsverbund Öffentliche Gesundheit: Evidenztransfer im ÖGD durch neue Kooperations- und Qualifikationswege. https://www.akademie-oegw.de/die-akademie/projekte/archiv/evidenzoegd. Zugegriffen: 14. Juli 2023

[21] Tricco AC, Lillie E, Zarin W, O’Brien KK, Colquhoun H, Levac D et al (2018) PRISMA Extension for Scoping Reviews (PRISMA-ScR): Checklist and Explanation. Ann Intern Med 169(7):467–473. 10.7326/M18-0850 30178033 10.7326/M18-0850

[22] Altgeld T (2023) New Public Health und ÖGD-Reformen: eine kritische Bestandsanalyse. Public Health Forum 31(4):250–253. 10.1515/pubhef-2023-0123

[23] Beirat Pakt ÖGD (2023) Multiprofessionalität ausbauen und fördern – für einen zukunftsfähigen ÖGD: Strukturelle und zukunftsorientierte Weiterentwicklung des Öffentlichen Gesundheitsdienstes. https://www.bundesgesundheitsministerium.de/fileadmin/Dateien/3_Downloads/O/OEGD/230515_BMG_4_Bericht_Beirat_Pakt_OeGD_bf.pdf. Zugegriffen: 11. Juni 2025

[24] Sachverständigenrat Gesundheit und Pflege (2023) Resilienz im Gesundheitswesen: Wege zur Bewältigung künftiger Krisen Gutachten 2023. https://www.svr-gesundheit.de/fileadmin/Gutachten/Gutachten_2023/Gesamtgutachten_ePDF_Final.pdf. Zugegriffen: 11. Juni 2025

[25] Hochschulen-Liste.de (2025) Deutsche Hochschulen nach Bundesland sortiert. https://hochschulen-liste.de/bundesland/. Zugegriffen: 11. Juni 2025

[26] Kuhn J (2024) 360, 377, 383: Wie viele Gesundheitsämter gibt es in Deutschland? https://scienceblogs.de/gesundheits-check/2024/06/13/360-377-383-wie-viele-gesundheitsaemter-gibt-es-in-deutschland/. Zugegriffen: 13. Juni 2025

[27] Statistisches Bundesamt (2024) Personal in Forschung und Entwicklung nach Bundesländern und Sektoren-Vollzeitäquivalent. https://www.destatis.de/DE/Themen/Gesellschaft-Umwelt/Bildung-Forschung-Kultur/Forschung-Entwicklung/Tabellen/fue-personal-bundeslaender-sektoren.html. Zugegriffen: 11. Juni 2025

[28] Bundesministerium für Forschung, Technologie und Raumfahrt (2023) Neue Ausschreibungen im EU4Health-Programm mit Bezug zu COVID-19. https://www.nksgesundheit.de/de/Neue-Ausschreibungen-im-EU4Health-Programm-mit-Bezug-zu-COVID-19-2938.html. Zugegriffen: 11. Juni 2025

[29] Gemeinsamer Bundesausschuss (2025) Förderbekanntmachung Versorgungsforschung zum themenspezifischen Bereich. https://innovationsfonds.g-ba.de/foerderbekanntmachungen/foerderbekanntmachung-versorgungsforschung-zum-themenspezifischen-bereich.52. Zugegriffen: 11. Juni 2025

[30] Bundesministerium für Foschung, Technologie und Raumfahrt (2023) Coronaviren im Fokus: Die BMBF-Forschungsförderung – DLR Gesundheitsforschung. https://www.gesundheitsforschung-bmbf.de/de/coronaviren-im-fokus-die-bmbf-forschungsforderung-15598.php. Zugegriffen: 11. Juni 2025

[31] Länderoffene Projektgruppe „Leitbild ÖGD“ (2018) Konsens der länderoffenen Arbeitsgruppe zu einem Leitbild für einen modernen Öffentlichen Gesundheitsdienst. Gesundheitswesen 80:679–681. 10.1055/a-0664-9349

[32] Bundesministerium für Gesundheit (BMG) (2025) Personalaufbau (Pakt für den ÖGD) | BMG. https://www.bundesgesundheitsministerium.de/themen/gesundheitswesen/pakt-fuer-den-oegd/personalaufbau-oegd.html. Zugegriffen: 11. Juni 2025

[33] Bundesministerium für Gesundheit (BMG) (2024) Personalaufwuchs im Rahmen des Paktes für den ÖGD bis Ende 2023. https://www.bundesgesundheitsministerium.de/fileadmin/Dateien/3_Downloads/O/OEGD/Personalaufwuchs_fuer_das_Jahr_2023.pdf. Zugegriffen: 11. Juni 2025

[34] Arnold L, Steinisch M, Kuehne A, Schwabe A, Jakubowski E, Scholten A et al (2025) Leitlinien im und für den Öffentlichen Gesundheitsdienst: Ergebnisse einer Onlinebefragung zu aktuellen Bedarfen aus der Praxis. Gesundheitswesen 87(1):57–61. 10.1055/a-2406-4786 39379022 10.1055/a-2406-4786 PMC11740218

[35] Arnold L, Bimczok S, Dragano N, Kietzmann A, Schäfer M, Schenuit G et al (2024) Pilotierung eines Trainee-Rotationsmodells zur Förderung kommunaler Wissenstransfer- und Kooperationsstrukturen. Erste Ergebnisse aus der Evaluation des EvidenzÖGD-Projektes. In: Der Öffentliche Gesundheitsdienst – Rückenwind für Gesundheit! 73. Thieme, Gesundheitswesen

[36] Akademie für Öffentliches Gesundheitswesen (AÖGW) (2025) EvidenzÖGD-Weiterentwicklung. Das Trainee-Rotationsprogramm Evidenz-ÖGD für Gesundheitsämter und Hochschulen. https://www.akademie-oegw.de/die-akademie/projekte/evidenzoegd2. Zugegriffen: 11. Juni 2025

[37] Universitätsklinikum Tübingen (2025) Evaluation PJ im ÖGD. https://www.medizin.uni-tuebingen.de/de/das-klinikum/einrichtungen/zentren/gesundheitswesen-und-versorgungsforschung/forschung/evaluation-pj. Zugegriffen: 11. Juni 2025

[38] Heinrich-Heine-Universität Düsseldorf (HHU) (2021) Lehre in Bewegung: Die Zukunft der Ausbildung im Öffentlichen Gesundheitsdienst. https://www.medizin.hhu.de/news-detailinformation/lehre-in-bewegung-die-zukunft-der-ausbildung-im-oeffentlichen-gesundheitsdienst. Zugegriffen: 11. Juni 2025

[39] Robert Koch-Institut (RKI) (2021) Postgraduiertenausbildung für angewandte Epidemiologie (PAE) am RKI: (Deutsches Feldepidemiologie-Trainingsprogramm, FETP): Abteilung für Infektionsepidemiologie am RKI. https://www.rki.de/DE/Institut/Das-RKI/Karriere/Ausbildungsprogramme/PAE/Infektepidem_Training.html. Zugegriffen: 23. Jan. 2025

[40] Kiefer S, Finke D, Kühne A, Arnold L, Weisshaar H, von Berenberg P et al (2025) Stärkung epidemiologischer Kompetenzen im kommunalen ÖGD: Erkenntnisse und Perspektiven aus dem FETP4ÖGD-Projekt. Gesundheitswesen 87(01):S 11–S11. 10.1055/s-0045-1801908

[41] World Health Organization (WHO) (2013) The Helsinki Statement on Health in All Policies. Helsinki: The 8th Global Conference on Health Promotion. https://www.who.int/healthpromotion/conferences/8gchp/en/. Zugegriffen: 10. Okt. 2019

