# Supplementary material for: Cooperation between science and practice in public health services: a systematic mapping from 2015 to 2024
Source: Bundesgesundheitsblatt Gesundheitsforschung Gesundheitsschutz. 2025 Dec 1;69(1):3–14. [Article in German] doi: 10.1007/s00103-025-04161-y (PMC12764607; doi:10.1007/s00103-025-04161-y)
Supplement: Supplementary file 1 — ESM 1: Suchbegriffe und -kombinationen in der systematischen Suche in wissenschaftlichen Datenbanken und über GoogleTM; ESM 2: Ausführliches Kodierschema zur Analyse der Kooperationen zwischen Wissenschaft und Praxis im ÖGD; ESM 3: Ausgefüllte PRISMA-ScR-Guideline(Preferred Reporting Items for Systematic reviews and Meta-Analyses extension for Scoping Reviews)-Checkliste; ESM 4: Ausführliche Ergebnistabelle des Kooperationsscreenings [file 103_2025_4161_MOESM1_ESM.pdf]

Zusätzliches Onlinematerial (ZOM)

ZOM 1: Suchbegriffe und -kombinationen in der systematischen Suche in wissenschaftlichen Datenbanken und über Google™ 2

ZOM 2: Ausführliches Kodierschema zur Analyse der Kooperationen zwischen Wissenschaft und Praxis im ÖGD 10

ZOM 3: Ausgefüllte PRISMA-ScR-Guideline (Preferred Reporting Items for Systematic reviews and Meta-Analyses extension for Scoping Reviews) Checkliste ...18

ZOM 4: Ausführliche Ergebnistabelle des Kooperationsscreenings...20

ZOM 1: Suchbegriffe und -kombinationen in der systematischen Suche in wissenschaftlichen Datenbanken und über Google™

Datenbank	*Suchdatum*	*Suchzeitraum*	*Suchstrang*	*Anzahl Treffer*
PubMed	*02.08.2022*	*2015-2022*	*Gesundheitsamt OR Gesundheitsämter OR Behörde für Gesundheit OR Behörden für Gesundheit OR Gesundheitsbehörde OR Gesundheitsbehörden OR Öffentlicher Gesundheitsdienst*	*189*
PubMed	*26.11.2024*	*2022-2024*	*Gesundheitsamt OR Gesundheitsämter OR Behörde für Gesundheit OR Behörden für Gesundheit OR Gesundheitsbehörde OR Gesundheitsbehörden OR Öffentlicher Gesundheitsdienst*	*96*
LIVIVO	*04.01.2023*	*2015-2022*	*IN = ("Gesundheitsamt" OR "Gesundheitsämter" OR "Behörde für Gesundheit" OR "Behörden für Gesundheit" OR "Gesundheitsbehörde" OR "Gesundheitsbehörden" OR "Öffentlicher Gesundheitsdienst")*	*169*
LIVIVO	*26.11.2024*	*2022-2024*	*IN = ("Gesundheitsamt" OR "Gesundheitsämter" OR "Behörde für Gesundheit" OR "Behörden für Gesundheit" OR "Gesundheitsbehörde" OR "Gesundheitsbehörden" OR "Öffentlicher Gesundheitsdienst")*	*22*
Google™	*26.07.2021*	*-*	*(Kooperation OR Zusammenarbeit OR Zusammenschluss OR Gemeinschaft OR Koalition OR Kollaboration OR Partnerschaft OR Konzept OR Programm OR Projekt) (Public Health OR Gesundheitswissenschaft OR Wissenschaft OR Forschung OR Studie) (ÖGD OR Öffentlicher Gesundheitsdienst OR Gesundheitsdienst OR Gesundheitsamt OR Kommune OR Gemeinde)*	*120*
Google™	*29.07.2021*	*-*	*(Kooperation OR Zusammenarbeit OR Zusammenschluss OR Gemeinschaft OR Koalition OR Kollaboration OR Partnerschaft OR Konzept OR Programm OR Projekt) (Public Health OR Gesundheitswissenschaft OR Wissenschaft OR Forschung OR Studie) (ÖGD OR Öffentlicher Gesundheitsdienst OR Gesundheitsdienst OR Gesundheitsamt OR Kommune OR Gemeinde) (Schuleingangsuntersuchung OR Einschulungsuntersuchung OR ESU OR SEU OR Schulneulingsuntersuchung OR KJGD OR Kinder- und Jugendgesundheitsdienst)*	*120*
Google™	*13.08.2021*	*-*	*(Kooperation OR Zusammenarbeit OR Kollaboration OR Partnerschaft OR Konzept OR Projekt OR Studie) (Public Health OR Gesundheitswissenschaft OR Wissenschaft OR Forschung OR Wissenschaftliche Stärkung OR Universität) (ÖGD OR Öffentlicher Gesundheitsdienst OR Gesundheitsbehörde OR Gesundheitsamt OR Kommune OR Kommunal OR Bevölkerungsgesundheit OR Öffentliche Gesundheit)*	*122*

Google™	*25.08.2021*	-	*(Vernetzung OR Verknüpfung OR Netzwerk OR Kooperationsnetzwerk OR Bündnis OR Allianz OR Evaluation) (Public Health OR Gesundheitswissenschaft OR Wissenschaft OR Forschung OR Wissenschaftliche Stärkung OR Universität) (ÖGD OR Öffentlicher Gesundheitsdienst OR Gesundheitsbehörde OR Gesundheitsamt OR Kommune OR Kommunal OR Bevölkerungsgesundheit OR Öffentliche Gesundheit)*	*134*
Google™	*27.08.2021*	-	*(Kooperation OR Zusammenarbeit OR Kollaboration OR Partnerschaft OR Konzept OR Projekt OR Studie) (Public Health OR Gesundheitswissenschaft OR Wissenschaft OR Forschung OR Wissenschaftliche Stärkung OR Universität) (ÖGD OR Öffentlicher Gesundheitsdienst OR Gesundheitsbehörde OR Gesundheitsamt OR Kommune OR Kommunal OR Bevölkerungsgesundheit OR Öffentliche Gesundheit) (Schuleingangsuntersuchung OR Einschulungsuntersuchung OR ESU OR SEU OR Schulneulingsuntersuchung OR KJGD OR Jugendgesundheitsdienst)*	*119*
Google™	*30.08.2021*	-	*(Vernetzung OR Verknüpfung OR Netzwerk OR Kooperationsnetzwerk OR Bündnis OR Allianz OR Evaluation) (Public Health OR Gesundheitswissenschaft OR Wissenschaft OR Forschung OR Wissenschaftliche Stärkung OR Universität) (ÖGD OR Öffentlicher Gesundheitsdienst OR Gesundheitsbehörde OR Gesundheitsamt OR Kommune OR Kommunal OR Bevölkerungsgesundheit OR Öffentliche Gesundheit) (Schuleingangsuntersuchung OR Einschulungsuntersuchung OR ESU OR SEU OR Schulneulingsuntersuchung OR KJGD OR Jugendgesundheitsdienst)*	*129*
Google™	*30.08.2021*	-	*(Kooperation OR Zusammenarbeit OR Kollaboration OR Partnerschaft OR Konzept OR Projekt OR Studie) (Public Health OR Gesundheitswissenschaft OR Wissenschaft OR Forschung OR Wissenschaftliche Stärkung OR Universität) (ÖGD OR Öffentlicher Gesundheitsdienst OR Gesundheitsbehörde OR Gesundheitsamt OR Kommune OR Kommunal OR Bevölkerungsgesundheit OR Öffentliche Gesundheit) (Infektionsschutz OR Infektionsepidemiologie OR Seroprävalenz)*	*102*
Google™	*30.08.2021*	-	*(Vernetzung OR Verknüpfung OR Netzwerk OR Kooperationsnetzwerk OR Bündnis OR Allianz OR Evaluation) (Public Health OR Gesundheitswissenschaft OR Wissenschaft OR Forschung OR Wissenschaftliche Stärkung OR Universität) (ÖGD OR Öffentlicher Gesundheitsdienst OR Gesundheitsbehörde OR Gesundheitsamt OR Kommune OR Kommunal OR Bevölkerungsgesundheit OR Öffentliche Gesundheit) (Infektionsschutz OR Infektionsepidemiologie OR Seroprävalenz)*	*99*
Google™	*31.08.2021*	-	*(Kooperation OR Zusammenarbeit OR Kollaboration OR Partnerschaft OR Konzept OR Projekt OR Studie) (Public Health OR Gesundheitswissenschaft OR Wissenschaft OR Forschung OR Wissenschaftliche Stärkung OR Universität) (ÖGD OR Öffentlicher Gesundheitsdienst OR Gesundheitsbehörde OR Gesundheitsamt OR Kommune OR Kommunal OR*	*117*

			Bevölkerungsgesundheit OR Öffentliche Gesundheit) (Gesundheitsberichterstattung OR GBE OR Gesundheitsmonitoring OR Surveillance OR Gesundheitsindikatoren OR Koordination OR Planung)	
Google™	*31.08.2021*	*-*	*(Vernetzung OR Verknüpfung OR Netzwerk OR Kooperationsnetzwerk OR Bündnis OR Allianz OR Evaluation) (Public Health OR Gesundheitswissenschaft OR Wissenschaft OR Forschung OR Wissenschaftliche Stärkung OR Universität) (ÖGD OR Öffentlicher Gesundheitsdienst OR Gesundheitsbehörde OR Gesundheitsamt OR Kommune OR Kommunal OR Bevölkerungsgesundheit OR Öffentliche Gesundheit) (Gesundheitsberichterstattung OR GBE OR Gesundheitsmonitoring OR Surveillance OR Gesundheitsindikatoren OR Koordination OR Planung)*	*113*
Google™	*31.08.2021*	*-*	*(Kooperation OR Zusammenarbeit OR Kollaboration OR Partnerschaft OR Konzept OR Projekt OR Studie) (Public Health OR Gesundheitswissenschaft OR Wissenschaft OR Forschung OR Wissenschaftliche Stärkung OR Universität) (ÖGD OR Öffentlicher Gesundheitsdienst OR Gesundheitsbehörde OR Gesundheitsamt OR Kommune OR Kommunal OR Bevölkerungsgesundheit OR Öffentliche Gesundheit) (Gesundheitsförderung OR Prävention OR Fürsorge)*	*128*
Google™	*31.08.2021*	*-*	*(Vernetzung OR Verknüpfung OR Netzwerk OR Kooperationsnetzwerk OR Bündnis OR Allianz OR Evaluation) (Public Health OR Gesundheitswissenschaft OR Wissenschaft OR Forschung OR Wissenschaftliche Stärkung OR Universität) (ÖGD OR Öffentlicher Gesundheitsdienst OR Gesundheitsbehörde OR Gesundheitsamt OR Kommune OR Kommunal OR Bevölkerungsgesundheit OR Öffentliche Gesundheit) (Gesundheitsförderung OR Prävention OR Fürsorge)*	*127*
Google™	*26.08.2021*	*-*	*(Qualifikation OR Kompetenz OR Ausbildung OR Weiterbildung OR Fortbildung OR Fähigkeit OR Fachwissen OR Trainee) (Public Health OR Gesundheitswissenschaft OR Wissenschaft OR Forschung OR Wissenschaftliche Stärkung OR Universität) (ÖGD OR Öffentlicher Gesundheitsdienst OR Gesundheitsbehörde OR Gesundheitsamt OR Kommune OR Kommunal OR Bevölkerungsgesundheit OR Öffentliche Gesundheit)*	*108*
Google™	*26.08.2021*	*-*	*(Curriculum OR Postgraduate OR PhD OR Doktor OR Programm OR Master OR Workforce OR Lernziele) (Public Health OR Gesundheitswissenschaft OR Wissenschaft OR Forschung OR Wissenschaftliche Stärkung OR Universität) (ÖGD OR Öffentlicher Gesundheitsdienst OR Gesundheitsbehörde OR Gesundheitsamt OR Kommune OR Kommunal OR Bevölkerungsgesundheit OR Öffentliche Gesundheit)*	*70*
Google™	*31.08.2021*	*-*	*(Qualifikation OR Kompetenz OR Ausbildung OR Weiterbildung OR Fortbildung OR Fähigkeit OR Fachwissen OR Trainee) (Public Health OR Gesundheitswissenschaft OR Wissenschaft OR Forschung OR Wissenschaftliche Stärkung OR Universität)*	*123*

			(ÖGD OR Öffentlicher Gesundheitsdienst OR Gesundheitsbehörde OR Gesundheitsamt OR Kommune OR Kommunal OR Bevölkerungsgesundheit OR Öffentliche Gesundheit) (Schuleingangsuntersuchung OR Einschulungsuntersuchung OR ESU OR SEU OR Schulneulingsuntersuchung OR KJGD OR Jugendgesundheitsdienst)	
Google™	*31.08.2021*	-	*(Curriculum OR Postgraduate OR PhD OR Doktor OR Programm OR Master OR Workforce OR Lernziele) (Public Health OR Gesundheitswissenschaft OR Wissenschaft OR Forschung OR Wissenschaftliche Stärkung OR Universität) (ÖGD OR Öffentlicher Gesundheitsdienst OR Gesundheitsbehörde OR Gesundheitsamt OR Kommune OR Kommunal OR Bevölkerungsgesundheit OR Öffentliche Gesundheit) (Schuleingangsuntersuchung OR Einschulungsuntersuchung OR ESU OR SEU OR Schulneulingsuntersuchung OR KJGD OR Jugendgesundheitsdienst)*	*119*
Google™	*01.09.2021*	-	*(Qualifikation OR Kompetenz OR Ausbildung OR Weiterbildung OR Fortbildung OR Fähigkeit OR Fachwissen OR Trainee) (Public Health OR Gesundheitswissenschaft OR Wissenschaft OR Forschung OR Wissenschaftliche Stärkung OR Universität) (ÖGD OR Öffentlicher Gesundheitsdienst OR Gesundheitsbehörde OR Gesundheitsamt OR Kommune OR Kommunal OR Bevölkerungsgesundheit OR Öffentliche Gesundheit) (Infektionsschutz OR Infektionsepidemiologie OR Seroprävalenz)*	*101*
Google™	*01.09.2021*	-	*(Curriculum OR Postgraduate OR PhD OR Doktor OR Programm OR Master OR Workforce OR Lernziele) (Public Health OR Gesundheitswissenschaft OR Wissenschaft OR Forschung OR Wissenschaftliche Stärkung OR Universität) (ÖGD OR Öffentlicher Gesundheitsdienst OR Gesundheitsbehörde OR Gesundheitsamt OR Kommune OR Kommunal OR Bevölkerungsgesundheit OR Öffentliche Gesundheit) (Infektionsschutz OR Infektionsepidemiologie OR Seroprävalenz)*	*96*
Google™	*01.09.2021*	-	*(Qualifikation OR Kompetenz OR Ausbildung OR Weiterbildung OR Fortbildung OR Fähigkeit OR Fachwissen OR Trainee) (Public Health OR Gesundheitswissenschaft OR Wissenschaft OR Forschung OR Wissenschaftliche Stärkung OR Universität) (ÖGD OR Öffentlicher Gesundheitsdienst OR Gesundheitsbehörde OR Gesundheitsamt OR Kommune OR Kommunal OR Bevölkerungsgesundheit OR Öffentliche Gesundheit) (Gesundheitsberichterstattung OR GBE OR Gesundheitsmonitoring OR Surveillance OR Gesundheitsindikatoren OR Koordination OR Planung)*	*117*
Google™	*01.09.2021*	-	*(Curriculum OR Postgraduate OR PhD OR Doktor OR Programm OR Master OR Workforce OR Lernziele) (Public Health OR Gesundheitswissenschaft OR Wissenschaft OR Forschung OR Wissenschaftliche Stärkung OR Universität) (ÖGD OR Öffentlicher Gesundheitsdienst OR Gesundheitsbehörde OR Gesundheitsamt OR Kommune OR Kommunal OR*	*100*

			Bevölkerungsgesundheit OR Öffentliche Gesundheit) (Gesundheitsberichterstattung OR GBE OR Gesundheitsmonitoring OR Surveillance OR Gesundheitsindikatoren OR Koordination OR Planung)	
Google™	*01.09.2021*	*-*	*(Qualifikation OR Kompetenz OR Ausbildung OR Weiterbildung OR Fortbildung OR Fähigkeit OR Fachwissen OR Trainee) (Public Health OR Gesundheitswissenschaft OR Wissenschaft OR Forschung OR Wissenschaftliche Stärkung OR Universität) (ÖGD OR Öffentlicher Gesundheitsdienst OR Gesundheitsbehörde OR Gesundheitsamt OR Kommune OR Kommunal OR Bevölkerungsgesundheit OR Öffentliche Gesundheit) (Gesundheitsförderung OR Prävention OR Fürsorge)*	*124*
Google™	*01.09.2021*	*-*	*(Curriculum OR Postgraduate OR PhD OR Doktor OR Programm OR Master OR Workforce OR Lernziele) (Public Health OR Gesundheitswissenschaft OR Wissenschaft OR Forschung OR Wissenschaftliche Stärkung OR Universität) (ÖGD OR Öffentlicher Gesundheitsdienst OR Gesundheitsbehörde OR Gesundheitsamt OR Kommune OR Kommunal OR Bevölkerungsgesundheit OR Öffentliche Gesundheit) (Gesundheitsförderung OR Prävention OR Fürsorge)*	*116*
Google™	*04.11.2024*	*-*	*(Kooperation OR Zusammenarbeit OR Kollaboration OR Partnerschaft OR Konzept OR Projekt OR Studie) (Public Health OR Gesundheitswissenschaft OR Wissenschaft OR Forschung OR Wissenschaftliche Stärkung OR Universität) (ÖGD OR Öffentlicher Gesundheitsdienst OR Gesundheitsbehörde OR Gesundheitsamt OR Kommune OR Kommunal OR Bevölkerungsgesundheit OR Öffentliche Gesundheit)*	*159*
Google™	*04.11.2024*	*-*	*(Vernetzung OR Verknüpfung OR Netzwerk OR Kooperationsnetzwerk OR Bündnis OR Allianz OR Evaluation) (Public Health OR Gesundheitswissenschaft OR Wissenschaft OR Forschung OR Wissenschaftliche Stärkung OR Universität) (ÖGD OR Öffentlicher Gesundheitsdienst OR Gesundheitsbehörde OR Gesundheitsamt OR Kommune OR Kommunal OR Bevölkerungsgesundheit OR Öffentliche Gesundheit)*	*165*
Google™	*05.11.2024*	*-*	*(Kooperation OR Zusammenarbeit OR Kollaboration OR Partnerschaft OR Konzept OR Projekt OR Studie) (Public Health OR Gesundheitswissenschaft OR Wissenschaft OR Forschung OR Wissenschaftliche Stärkung OR Universität) (ÖGD OR Öffentlicher Gesundheitsdienst OR Gesundheitsbehörde OR Gesundheitsamt OR Kommune OR Kommunal OR Bevölkerungsgesundheit OR Öffentliche Gesundheit) (Schuleingangsuntersuchung OR Einschulungsuntersuchung OR ESU OR SEU OR Schulneulingsuntersuchung OR KJGD OR Jugendgesundheitsdienst)*	*75*
Google™	*05.11.2024*	*-*	*(Vernetzung OR Verknüpfung OR Netzwerk OR Kooperationsnetzwerk OR Bündnis OR Allianz OR Evaluation) (Public Health OR Gesundheitswissenschaft OR Wissenschaft OR Forschung OR Wissenschaftliche Stärkung OR Universität) (ÖGD OR*	*72*

			Öffentlicher Gesundheitsdienst OR Gesundheitsbehörde OR Gesundheitsamt OR Kommune OR Kommunal OR Bevölkerungsgesundheit OR Öffentliche Gesundheit) (Schuleingangsuntersuchung OR Einschulungsuntersuchung OR ESU OR SEU OR Schulneulingsuntersuchung OR KJGD OR Jugendgesundheitsdienst)	
Google™	*11.11.2024*	*-*	*(Kooperation OR Zusammenarbeit OR Kollaboration OR Partnerschaft OR Konzept OR Projekt OR Studie) (Public Health OR Gesundheitswissenschaft OR Wissenschaft OR Forschung OR Wissenschaftliche Stärkung OR Universität) (ÖGD OR Öffentlicher Gesundheitsdienst OR Gesundheitsbehörde OR Gesundheitsamt OR Kommune OR Kommunal OR Bevölkerungsgesundheit OR Öffentliche Gesundheit) (Infektionsschutz OR Infektionsepidemiologie OR Seroprävalenz)*	*145*
Google™	*11.11.2024*	*-*	*(Vernetzung OR Verknüpfung OR Netzwerk OR Kooperationsnetzwerk OR Bündnis OR Allianz OR Evaluation) (Public Health OR Gesundheitswissenschaft OR Wissenschaft OR Forschung OR Wissenschaftliche Stärkung OR Universität) (ÖGD OR Öffentlicher Gesundheitsdienst OR Gesundheitsbehörde OR Gesundheitsamt OR Kommune OR Kommunal OR Bevölkerungsgesundheit OR Öffentliche Gesundheit) (Infektionsschutz OR Infektionsepidemiologie OR Seroprävalenz)*	*142*
Google™	*11.11.2024*	*-*	*(Kooperation OR Zusammenarbeit OR Kollaboration OR Partnerschaft OR Konzept OR Projekt OR Studie) (Public Health OR Gesundheitswissenschaft OR Wissenschaft OR Forschung OR Wissenschaftliche Stärkung OR Universität) (ÖGD OR Öffentlicher Gesundheitsdienst OR Gesundheitsbehörde OR Gesundheitsamt OR Kommune OR Kommunal OR Bevölkerungsgesundheit OR Öffentliche Gesundheit) (Gesundheitsberichterstattung OR GBE OR Gesundheitsmonitoring OR Surveillance OR Gesundheitsindikatoren OR Koordination OR Planung)*	*163*
Google™	*11.11.2024*	*-*	*(Vernetzung OR Verknüpfung OR Netzwerk OR Kooperationsnetzwerk OR Bündnis OR Allianz OR Evaluation) (Public Health OR Gesundheitswissenschaft OR Wissenschaft OR Forschung OR Wissenschaftliche Stärkung OR Universität) (ÖGD OR Öffentlicher Gesundheitsdienst OR Gesundheitsbehörde OR Gesundheitsamt OR Kommune OR Kommunal OR Bevölkerungsgesundheit OR Öffentliche Gesundheit) (Gesundheitsberichterstattung OR GBE OR Gesundheitsmonitoring OR Surveillance OR Gesundheitsindikatoren OR Koordination OR Planung)*	*160*
Google™	*11.11.2024*	*-*	*(Kooperation OR Zusammenarbeit OR Kollaboration OR Partnerschaft OR Konzept OR Projekt OR Studie) (Public Health OR Gesundheitswissenschaft OR Wissenschaft OR Forschung OR Wissenschaftliche Stärkung OR Universität) (ÖGD OR Öffentlicher Gesundheitsdienst OR Gesundheitsbehörde OR Gesundheitsamt OR Kommune OR Kommunal OR Bevölkerungsgesundheit OR Öffentliche Gesundheit) (Gesundheitsförderung OR Prävention OR Fürsorge)*	*157*

Google™	*11.11.2024*	*-*	*(Vernetzung OR Verknüpfung OR Netzwerk OR Kooperationsnetzwerk OR Bündnis OR Allianz OR Evaluation) (Public Health OR Gesundheitswissenschaft OR Wissenschaft OR Forschung OR Wissenschaftliche Stärkung OR Universität) (ÖGD OR Öffentlicher Gesundheitsdienst OR Gesundheitsbehörde OR Gesundheitsamt OR Kommune OR Kommunal OR Bevölkerungsgesundheit OR Öffentliche Gesundheit) (Gesundheitsförderung OR Prävention OR Fürsorge)*	*160*
Google™	*11.11.2024*	*-*	*(Qualifikation OR Kompetenz OR Ausbildung OR Weiterbildung OR Fortbildung OR Fähigkeit OR Fachwissen OR Trainee) (Public Health OR Gesundheitswissenschaft OR Wissenschaft OR Forschung OR Wissenschaftliche Stärkung OR Universität) (ÖGD OR Öffentlicher Gesundheitsdienst OR Gesundheitsbehörde OR Gesundheitsamt OR Kommune OR Kommunal OR Bevölkerungsgesundheit OR Öffentliche Gesundheit)*	*160*
Google™	*11.11.2024*	*-*	*(Curriculum OR Postgraduate OR PhD OR Doktor OR Programm OR Master OR Workforce OR Lernziele) (Public Health OR Gesundheitswissenschaft OR Wissenschaft OR Forschung OR Wissenschaftliche Stärkung OR Universität) (ÖGD OR Öffentlicher Gesundheitsdienst OR Gesundheitsbehörde OR Gesundheitsamt OR Kommune OR Kommunal OR Bevölkerungsgesundheit OR Öffentliche Gesundheit)*	*155*
Google™	*11.11.2024*	*-*	*(Qualifikation OR Kompetenz OR Ausbildung OR Weiterbildung OR Fortbildung OR Fähigkeit OR Fachwissen OR Trainee) (Public Health OR Gesundheitswissenschaft OR Wissenschaft OR Forschung OR Wissenschaftliche Stärkung OR Universität) (ÖGD OR Öffentlicher Gesundheitsdienst OR Gesundheitsbehörde OR Gesundheitsamt OR Kommune OR Kommunal OR Bevölkerungsgesundheit OR Öffentliche Gesundheit) (Schuleingangsuntersuchung OR Einschulungsuntersuchung OR ESU OR SEU OR Schulneulingsuntersuchung OR KJGD OR Jugendgesundheitsdienst)*	*70*
Google™	*11.11.2024*	*-*	*(Curriculum OR Postgraduate OR PhD OR Doktor OR Programm OR Master OR Workforce OR Lernziele) (Public Health OR Gesundheitswissenschaft OR Wissenschaft OR Forschung OR Wissenschaftliche Stärkung OR Universität) (ÖGD OR Öffentlicher Gesundheitsdienst OR Gesundheitsbehörde OR Gesundheitsamt OR Kommune OR Kommunal OR Bevölkerungsgesundheit OR Öffentliche Gesundheit) (Schuleingangsuntersuchung OR Einschulungsuntersuchung OR ESU OR SEU OR Schulneulingsuntersuchung OR KJGD OR Jugendgesundheitsdienst)*	*76*
Google™	*11.11.2024*	*-*	*(Qualifikation OR Kompetenz OR Ausbildung OR Weiterbildung OR Fortbildung OR Fähigkeit OR Fachwissen OR Trainee) (Public Health OR Gesundheitswissenschaft OR Wissenschaft OR Forschung OR Wissenschaftliche Stärkung OR Universität)*	*137*

			(ÖGD OR Öffentlicher Gesundheitsdienst OR Gesundheitsbehörde OR Gesundheitsamt OR Kommune OR Kommunal OR Bevölkerungsgesundheit OR Öffentliche Gesundheit) (Infektionsschutz OR Infektionsepidemiologie OR Seroprävalenz)	
Google™	*11.11.2024*	-	*(Curriculum OR Postgraduate OR PhD OR Doktor OR Programm OR Master OR Workforce OR Lernziele) (Public Health OR Gesundheitswissenschaft OR Wissenschaft OR Forschung OR Wissenschaftliche Stärkung OR Universität) (ÖGD OR Öffentlicher Gesundheitsdienst OR Gesundheitsbehörde OR Gesundheitsamt OR Kommune OR Kommunal OR Bevölkerungsgesundheit OR Öffentliche Gesundheit) (Infektionsschutz OR Infektionsepidemiologie OR Seroprävalenz)*	*132*
Google™	*11.11.2024*	-	*(Qualifikation OR Kompetenz OR Ausbildung OR Weiterbildung OR Fortbildung OR Fähigkeit OR Fachwissen OR Trainee) (Public Health OR Gesundheitswissenschaft OR Wissenschaft OR Forschung OR Wissenschaftliche Stärkung OR Universität) (ÖGD OR Öffentlicher Gesundheitsdienst OR Gesundheitsbehörde OR Gesundheitsamt OR Kommune OR Kommunal OR Bevölkerungsgesundheit OR Öffentliche Gesundheit) (Gesundheitsberichterstattung OR GBE OR Gesundheitsmonitoring OR Surveillance OR Gesundheitsindikatoren OR Koordination OR Planung)*	*156*
Google™	*11.11.2024*	-	*(Curriculum OR Postgraduate OR PhD OR Doktor OR Programm OR Master OR Workforce OR Lernziele) (Public Health OR Gesundheitswissenschaft OR Wissenschaft OR Forschung OR Wissenschaftliche Stärkung OR Universität) (ÖGD OR Öffentlicher Gesundheitsdienst OR Gesundheitsbehörde OR Gesundheitsamt OR Kommune OR Kommunal OR Bevölkerungsgesundheit OR Öffentliche Gesundheit) (Gesundheitsberichterstattung OR GBE OR Gesundheitsmonitoring OR Surveillance OR Gesundheitsindikatoren OR Koordination OR Planung)*	*144*
Google™	*11.11.2024*	-	*(Qualifikation OR Kompetenz OR Ausbildung OR Weiterbildung OR Fortbildung OR Fähigkeit OR Fachwissen OR Trainee) (Public Health OR Gesundheitswissenschaft OR Wissenschaft OR Forschung OR Wissenschaftliche Stärkung OR Universität) (ÖGD OR Öffentlicher Gesundheitsdienst OR Gesundheitsbehörde OR Gesundheitsamt OR Kommune OR Kommunal OR Bevölkerungsgesundheit OR Öffentliche Gesundheit) (Gesundheitsförderung OR Prävention OR Fürsorge)*	*152*
Google™	*11.11.2024*	-	*(Curriculum OR Postgraduate OR PhD OR Doktor OR Programm OR Master OR Workforce OR Lernziele) (Public Health OR Gesundheitswissenschaft OR Wissenschaft OR Forschung OR Wissenschaftliche Stärkung OR Universität) (ÖGD OR Öffentlicher Gesundheitsdienst OR Gesundheitsbehörde OR Gesundheitsamt OR Kommune OR Kommunal OR Bevölkerungsgesundheit OR Öffentliche Gesundheit) (Gesundheitsförderung OR Prävention OR Fürsorge)*	*145*

ZOM 2: Ausführliches Kategoriensystem zur Analyse der Kooperationen zwischen Wissenschaft und Praxis im ÖGD

Kategorie/ Auswertungsparameter	Unterkategorie	Kürzel zur Kodierung der Unterkategorie	Hinweis zur Kodierung
**ID**	-	[Nummer: 1-X]	Einzigartige Nummer zur Identifikation (Identifier); jede ID darf nur einmal vergeben sein. Neue Beiträge werden fortlaufend weiter nummeriert
**Quelle**	Kongress-Screening	[Kongresskürzel]	Jedes Kongresskürzel weißt auf einen Kongress hin. In manchen Jahren haben Kongresse von unterschiedlichen Fachgesellschaften gemeinsam, teilweise getrennt voneinander stattgefunden. Kongresskürzel an anderer Stelle vermerkt.
	Google Screening	[Suchkombination Google-Screening]	Bezeichnung des Suchdurchlaufs (siehe Übersicht der Suchdurchläufe)
	Snowballing	Snowballing	Hinweis darauf, dass die Kooperation über das Snowballing identifiziert wurde
	Projektnetzwerk	Projektnetzwerk	Hinweis darauf, dass die Kooperation über das Projektnetzwerk identifiziert wurde
	Online-Survey	Online-Survey	Hinweis darauf, dass die Kooperation über den Online-Survey identifiziert wurde
	Wissenschaftliche Datenbanken	[Datenbank]	Hinweis darauf, dass die Kooperation über das Screening in wissenschaftlichen Datenbanken identifiziert wurde
**Jahr**	-	[Jahreszahl]	Jahr, in welchem die Kooperation stattfand, bzw. bei mehrjährigen Kooperationen, in welchem Jahr sie startete
**Titel**	-	[Text]	Originaltitel des Beitrags (Titel so, wie er unter dem Link zu finden ist)
**Kooperationsform**	Unbefristete Kooperation	A	Langfristige Zusammenarbeit ohne festes Enddatum, die häufig nur zu bestimmten Anlässen / in bestimmten Zyklen aktiv werden (z. B. Netzwerke, Forschungsverbünde, Fach- oder Arbeitsgruppen)
	Projektbezogene Kooperation	B	Zeitlich begrenzte, zielgerichtete Zusammenarbeit im Rahmen eines Projekts, die mit dem Abschluss des Projektzeitraums oder nach Erreichen der Meilensteine endet. Sie gliedern sich oft in Arbeitspakete, können langfristige Kooperationen initiieren und Veröffentlichungen beinhalten; Eine Projektkooperation kann die Grundlage für die Entstehung einer etablierten Kooperation sein. Häufig beinhaltet ein Projekt mehrere Veröffentlichungen und mindestens einen gemeinsamen Projektbericht
	Publikationsbezogen e Kooperation	C	Zeitlich begrenzte Zusammenarbeit, deren Ergebnisse gemeinsam veröffentlicht werden

	Veranstaltungsbezogene Kooperation	D	Einmalige, zeitlich begrenzte Zusammenarbeit zur Planung und Durchführung einer spezifischen Veranstaltung (z. B. Vortrag, Workshop)
**Netzwerk-/ Projekttitel**	-	_Text_	Titel des Projektes oder des Netzwerkes, auf welches sich der Beitrag bezieht (falls die Kooperation Teil eines solchen ist vorhanden); Ein Beitrag kann sich auch auf mehrere oder auf gar kein Projekt/Netzwerk beziehen
**Zeitraum**	-	[Jahreszahlen des Start- und Endpunktes der Kooperation]	Zeitraum, in welchem das Projekt umgesetzt wurde, angegeben in Jahreszahlen; Sollte das Projekt noch nicht beendet sein, wird das Startdatum notiert und die Laufzeit bis heute eingetragen (z.B. „2018 - heute“)
**Kooperationsdauer**	-	[Zahl]	Dauer der Kooperation in Jahren, wobei Kooperationsbeginn und -abschluss jeweils als volles Jahr gewertet werden (Beispiel: Wenn die Kooperation von 2018-2021 ging, wird hier eine 4 notiert, da Umsetzung in 2018, 19, 20, 21.)
**Kooperationsstatus**	Abgeschlossen	Ja	Die Kooperation ist bereits abgeschlossen (Bezieht sich auf den Zeitpunkt 12/2024)
	Laufend	Nein	Die Kooperation ist noch nicht abgeschlossen (Bezieht sich auf den Zeitpunkt 12/2024)
**Publikationsform**	Poster(-präsentation)	P	Konzeption / Präsentation eines Posters in Kooperation: Entwicklung und Präsentation eines (wissenschaftlichen) Posters in Zusammenarbeit mit anderen, oft auf Konferenzen oder Fachveranstaltungen. Dient der kompakten Darstellung von Forschungsergebnissen oder Projekten.
	Workshop	W	Konzeption / Durchführung eines Workshops in Kooperation: Planung und Durchführung eines interaktiven Workshops in Kooperation mit anderen. Teilnehmende werden aktiv in das Thema eingebunden, um Wissen zu vermitteln oder Problemlösungen zu erarbeiten
	Vortrag	V	Konzeption / Durchführung eines Vortrags in Kooperation: Konzeption und Präsentation eines wissenschaftlichen oder fachlichen Vortrags in Kooperation, oft im Rahmen von Konferenzen, Tagungen oder anderen Fachveranstaltungen
	Fachforum, Symposium	F	Konzeption / Durchführung eines Fachforums / Symposiums in Kooperation: Organisation und Durchführung eines Fachforums oder Symposiums gemeinsam mit anderen Fachleuten. Dabei steht der wissenschaftliche Austausch im Mittelpunkt, oft mit mehreren Vorträgen und Diskussionen.
	Seminar	Se	Konzeption / Durchführung eines Seminars in Kooperation: Planung und Leitung eines Seminars in Zusammenarbeit mit anderen, meist mit didaktischem Konzept zur Vermittlung von Fachwissen an eine bestimmte Zielgruppe, z. B. Studierende oder Fachkräfte.

	Podiumsdiskussion	PD	Konzeption / Durchführung / Teilnahme an einer Podiumsdiskussion in Kooperation: Beteiligung an einer Podiumsdiskussion als Teilnehmender oder Moderator:in, oft in Kooperation mit anderen Expert:innen. Themen werden aus verschiedenen Perspektiven beleuchtet und diskutiert.
	Arbeitsgruppe	AG	Kooperation im Rahmen einer Arbeitsgruppe: Zusammenarbeit in einer organisierten Arbeitsgruppe, die sich mit einem spezifischen wissenschaftlichen oder fachlichen Thema befasst. Ergebnisse können in verschiedenen Formen publiziert werden.
	Abschlussarbeit	Ab	Kooperation im Rahmen einer akademischen Abschlussarbeit (Masterarbeit, Dissertation, Habilitation): Gemeinsame Arbeit an einer akademischen Abschlussarbeit, z. B. im Rahmen einer Masterarbeit, Dissertation oder Habilitation, oft mit externen Kooperationspartner:innen.
	Website	Web	Kooperation im Rahmen einer gemeinsamen Website: Gemeinsame Entwicklung und Pflege einer wissenschaftlichen oder themenspezifischen Website zur Veröffentlichung und Verbreitung von Informationen oder Forschungsergebnissen.
	Journal Article	J	Kooperation im Rahmen eines wissenschaftlichen Fachartikels: Kooperation bei der Erstellung eines wissenschaftlichen Fachartikels zur Veröffentlichung in einem wissenschaftlichen Journal. Umfasst typischerweise die gemeinsame Forschung, Analyse und das Verfassen des Artikels.
	Bericht	B	Kooperation im Rahmen eines Berichts (z.B. Projekt-, Gesundheits-, Fokus-, Evaluationsbericht): Zusammenarbeit an einem Bericht, z. B. Projekt-, Gesundheits-, Evaluations- oder Fokusbericht, der wissenschaftliche oder praxisrelevante Erkenntnisse zusammenfasst.
	Stellungnahme	St	Kooperation im Rahmen einer Stellungnahme: Gemeinsame Erstellung einer Stellungnahme zu einem fachlichen oder gesellschaftlichen Thema, oft zur Einflussnahme auf politische oder wissenschaftliche Diskussionen.
	Sonstiges	S	Das Publikationsformat kann nicht eindeutig zugeordnet werden: Publikationsform, die nicht eindeutig in eine der genannten Kategorien passt, aber dennoch im Rahmen einer Kooperation entstanden ist.
**Ansprechpersonen/ Autor:innen**	-	_Text_	Auflistung aller Autor:innen / Ansprechpersonen eines Beitrags; bei Projekten und Netzwerken sollten alle klar zu identifizierenden Ansprechpersonen aufgelistet werden. Bei Veröffentlichungen sollten alle Autor:innen aufgelistet werden. Erstautor:in sollte auch zuerst genannt werden. Falls möglich sollten diese Personen immer in folgendem Format aufgelistet werden: Erster Buchstabe des Vornamens + Nachname (Bei Doppelnamen werden beide Buchstaben verwendet); Trennung der Personen durch ein Semikolon + Leerzeichen; Beispiel: TJ Kühners; K Fehner; PB Bachmann; C Remmert<br>Alle überflüssigen Informationen (z.B. Institutionen und Städte hinter den Namen) entfernen
**Autor:innen (Original Info)**	-	_Text_	Originalinformationen zu allen Autor:innen / Ansprechpersonen. Hierbei sollen keine Änderungen am Text aus der ursprünglichen Affiliation vorgenommen werden (Strg+C und Str+F)
**Kooperierende Institutionen**	-	_Text_	Auflistung aller an der Kooperation beteiligten Institutionen. Beispiel: Robert Koch-Institut (RKI); Niedersächsisches Landesgesundheitsamt (NLGA); Heinrich-Heine-Universität Düsseldorf (HHU); Trennung der Akteur:innen/Institutionen durch ein Semikolon + Leerzeichen; Abkürzung in Klammern

			setzen (wenn vorhanden); Bezüglich der Schreibweise orientieren wir uns an den Webseiten der Institutionen
**Koordination bzw. Erstautor:innenschaft**	ÖGD - Kommunale Ebene	K	Die Projekt-/Netzwerkkoordination bzw. Erstautor:innenschaft liegt bei einer Person, die im ÖGD auf kommunaler Ebene tätig ist.
	ÖGD - Landesebene	L	Die Projekt-/Netzwerkkoordination bzw. Erstautor:innenschaft liegt bei einer Person, die im ÖGD auf Landesebene tätig ist.
	ÖGD - Bundesebene	B	Die Projekt-/Netzwerkkoordination bzw. Erstautor:innenschaft liegt bei einer Person, die im ÖGD auf Bundesebene tätig ist.
	Wissenschaft	W	Die Projekt-/Netzwerkkoordination bzw. Erstautor:innenschaft liegt bei einer Person von einer wissenschaftlichen Institution (z.B. Hochschule oder Universität)
	Sonstiges	X	Die Projekt-/Netzwerkkoordination bzw. Erstautor:innenschaft liegt weder bei einer ÖGD-Institution noch bei einer wissenschaftlichen Institution oder lässt sich nicht eindeutig zuordnen
**Co-Autor:innen**	-	_Zahl_	Nennung der Zahl weiterer (zusätzlich zu der/dem Erst:autor:in genannten Autor:innen
**ÖGD-Ebene der Institutionen**	Bundesebene	B	Es ist eine Institution des ÖGD von der Bundesebene (z.B. RKI, BMG; PEI; BZgA) beteiligt; Kodierung nach absteigender Ebene von B-K; Trennung bei Mehrfachkodierung mit Semikolon und Leerzeichen (z.B. B; K)
	Landesebene	L	Es ist eine Institution des ÖGD von der Landesebene (z.B. Landesgesundheitsämter; Landesgesundheitsministerien) beteiligt; Kodierung nach absteigender Ebene von B-K; Trennung bei Mehrfachkodierung mit Semikolon und Leerzeichen (z.B. B; K)
	Kommunale Ebene	K	Es ist eine Institution des ÖGD von der kommunalen Ebene (z.B. kommunales Gesundheitsamt, Fachdezernat Gesundheit) beteiligt; Kodierung nach absteigender Ebene von B-K; Trennung bei Mehrfachkodierung mit Semikolon und Leerzeichen (z.B. B; K)
	nicht eindeutig zuzuordnen/unklar	X	Die Institution lässt sich keiner ÖGD-Ebene eindeutig zuordnen; Kodierung nach absteigender Ebene von B-K; Trennung bei Mehrfachkodierung mit Semikolon und Leerzeichen (z.B. B; K)
**Umsetzungsebene**	Bundesebene	B	Die Kooperation wird auf nationaler Ebene umgesetzt, beispielsweise durch Zusammenarbeit mit Bundesbehörden, nationalen Forschungsinstituten oder übergreifenden wissenschaftlichen Netzwerken mit bundesweiter Reichweite; Labelling nach absteigender Ebene von B-K; Trennung bei Merfachlabelling mit Semikolon und Leerzeichen (z.B. B; K)
	Landesebene	L	Die Kooperation erfolgt innerhalb eines oder mehrerer Bundesländer, oft in Zusammenarbeit mit Landesbehörden oder regionalen Fachinstitutionen. Ziel kann z.B. die Entwicklung und Umsetzung von landesspezifischen Maßnahmen oder Forschungsprojekten sein; Labelling nach absteigender Ebene von B-K; Trennung bei Merfachlabelling mit Semikolon und Leerzeichen (z.B. B; K)

	Kommunale Ebene	K	Die Zusammenarbeit findet auf der Ebene einzelner Städte oder Gemeinden statt, oft mit kommunalen Entscheidungsträgern, um praxisnahe Lösungen zu entwickeln; Labelling nach absteigender Ebene von B-K; Trennung bei Merfachlabelling mit Semikolon und Leerzeichen (z.B. B; K)
	nicht eindeutig zuzuordnen/unklar	X	Die Umsetzungsebene der Kooperation lässt sich nicht klar einer der genannten Kategorien zuordnen oder bleibt unbestimmt, z.B. bei interdisziplinären Projekten mit gemischter Reichweite; Labelling nach absteigender Ebene von B-K; Trennung bei Merfachlabelling mit Semikolon und Leerzeichen (z.B. B; K)
**Thematische Einordnung**<br><br>Labelling von AD-X; Trennung bei Mehrfachlabelling mit Semikolon und Leerzeichen (z.B.: "AD; GP") - WICHTIG! --> Reihenfolge beachten	Amtsärztlicher Dienst/Gutachtenwesen (ÖGD-Arbeitsbereich)	AD	Kooperationen, bei denen der Amtsärztliche Dienst als Akteur agiert sowie Beiträge, bei denen der Amtsärztliche Dienst und seine Aufgaben selbst (u.a. personenbezogene Gutachten und Stellungnahmen nach gesetzlichen Vorschriften, Medizinalaufsicht inkl. Berufsaufsicht HeilpraktikerInnen, Registrierung der Todesbescheinigungen im Rahmen der Mortalitätsstatistik und Leichen- und Bestattungswesen inkl. Leichenschau vor Feuerbestattungen) Gegenstand der Forschung sind.
	Gesundheitsberichterstattung (GBE), Planung und Steuerung (ÖGD-Arbeitsbereich)	GBE	Kooperationen, bei denen Fragestellungen der GBE (z.B. Berichterstattung zum Gesundheitszustand der Bevölkerung/Gesundheitsversorgung/Gesundheitskosten/... zur Information von Politik, Wissenschaft und Fachöffentlichkeit) bearbeitet werden sowie solche, bei denen die GBE selbst Gegenstand der Forschung ist (z. B. Entwicklung der Guten Praxis GBE). Das beinhaltet unter anderem die Erwähnung von / Arbeit in Gesundheitskonferenzen (hier oftmals in enger Verzahnung mit der Gesundheitsförderung und Prävention --> ggf. doppelt codieren);<br>Schließt zudem alle Beiträge ein, die Kooperationen zwecks Planungs- , Steuerungs- und Abstimmungsprozessen beinhalten (z.B. Sozialplanung, Versorgungsplanung) (hier oftmals mit dem Ziel Gesundheit ressortübergreifend zu denken und zu planen – im Sinne von Health in all Policies (HiAP) --> vielfach in enger Verzahnung zu Gesundheitsförderung und Prävention --> ggf. doppelt codieren)
	Epidemiologie (ÖGD-Arbeitsbereich)	EPI	Kooperationen, die die Bearbeitung epidemiologischer Fragestellungen/Datenerhebungen (Erhebungen zu Häufigkeit von Krankheiten oder anderen gesundheitsbezogenen Merkmalen in (Gruppen) der Bevölkerung und Suche nach Beziehungen zu möglichen Ursachen) beinhalten.<br>Oftmals haben diese Beiträge einen speziellen Schwerpunkt, z.B.<br>- Sozialepidemiologie (--> ggf. doppelt codieren mit Soziale Ungleichheit)<br>- Infektionsepidemiologie (--> ggf. doppelt codieren mit Infektionsschutz & Hygiene)

	Gesundheitsförderung und Prävention (ÖGD-Arbeitsbereich)	GP	Kooperationen, die Fragestellungen der Gesundheitsförderung (Befähigung von Menschen zu mehr Kontrolle über ihre Gesundheit und die Verbesserung dieser) und der Prävention (Vermeidung, Verringerung/Abschwächung oder zeitliche Verschiebung von (Gesundheits-)Störungen) bearbeiten oder bei denen Gesundheitsförderung und Prävention selbst Gegenstand der Forschung sind. Gesundheitsförderung und Prävention sind oftmals Gegenstand der kommunalen Gesundheitskonferenzen (KGK) und dann ggf. in enger Verzahnung mit der GBE (--> ggf. doppelt codieren mit GBE)<br><br>NICHT: Kooperationen die ausschließlich auf Fürsorge, Beratung und Aufklärung fokussieren (ohne Setting- und Systemperspektive) --> diese werden mit X codiert<br><br>Oftmals haben diese Beiträge einen speziellen Schwerpunkt, z.B.<br>- Infektionsprävention (--> ggf. doppelt codieren mit Infektionsschutz & Hygiene)<br>- Gesundheitsförderung und Prävention im Kindes- und Jugendalter (--> ggf. doppelt codieren mit Kinder- und Jugendgesundheit)
	Infektionsschutz & Hygiene (ÖGD-Arbeitsbereich)	IH	Kooperationen, die Fragestellungen des Infektionsschutzes, z.B. bzgl. der Übertragung und Verbreitung eines Infektionserregers oder zu bevölkerungsbezogener Infektionsprävention (z.B. Überwachungssysteme, Ausbruchsuntersuchungen) bearbeiten oder bei denen der Infektionsschutz selbst Gegenstand der Forschung ist.
	Kinder- und Jugendgesundheit (ÖGD-Arbeitsbereich)	KJ	Kooperationen, bei denen der Kinder- und Jugendgesundheitsdienst (KJGD) als Akteur agiert sowie Beiträge, die sich der Bearbeitung von Fragestellungen der Kinder- und Jugendgesundheit (z.B. zu mentaler/physischer Gesundheit und Ernährung) widmen.
	Krisenmanagement (ÖGD-Arbeitsbereich)	KM	Kooperationen, die Fragestellungen des Krisenmanagements (systematischer Umgang mit gesundheitlichen Krisensituationen, inkl. der Identifikation, Analyse, Strategieentwicklung und Einleitung von Gegenmaßnahmen in Krisensituationen) bearbeiten, als auch Beiträge, bei denen das Krisenmanagement selbst Gegenstand der Forschung ist.
	Sozialpsychiatrie/Mentale Gesundheit (ÖGD-Arbeitsbereich)	SP	Kooperationen, bei denen der Sozialpsychiatrische Dienst als Akteur agiert sowie Beiträge, die sich der Bearbeitung von Fragestellungen der Sozialpsychiatrie widmen. Umfasst auch Beiträge, bei denen die Aufgaben des Sozialpsychiatrischen Dienstes selbst Forschungsgegenstand sind (z.B. Beratung und Hilfen für Menschen mit (Verdacht auf) Suchterkrankungen, oder für gerontopsychiatrisch erkrankte Menschen und für Menschen mit psychischen Erkrankungen, aufsuchende Angebote etc.)
	Sozialpharmazie (ÖGD-Arbeitsbereich)	SPh	Kooperationen, bei denen der Sozialpharmazeutische Dienst als Akteur agiert sowie Beiträge, die sich der Bearbeitung von Fragestellungen der Sozialpharmazie widmen.

	Umweltmedizin (ÖGD-Arbeitsbereich)	UM	Kooperationen, die Fragestellungen der Umweltmedizin (umweltbezogene Expositions- und Wirkungsermittlung sowie der Identifikation, Analyse, Strategieentwicklung und Einleitung von Gegenmaßnahmen bei umweltbedingten Gesundheitsrisiken (z.B. Trink-/Abwasseruntersuchungen, Luftverschmutzung, ...)) bearbeiten.
	Zahnmedizin (ÖGD-Arbeitsbereich)	Z	Kooperationen, die Fragestellungen der Zahngesundheit mit ÖGD-Bezug (sozial orientierte Zahn-, Mund- und Kieferheilkunde/Verbesserung der Mundgesundheit der Bevölkerung (Mundgesundheitsförderung)) bearbeiten.
	Migration und Gesundheit (ÖGD-Arbeitsbereich)	MG	Kooperationen, die Fragestellungen zur Gesundheit der Zielgruppe der legal und illegal zugewanderten Menschen mit Migrationsgeschichte bearbeiten.
	Soziale Ungleichheit (ÖGD-Arbeitsbereich)	SU	Kooperationen, die Fragestellungen der sozialen und gesundheitlichen Ungleichheit bearbeiten.<br>Das umfasst auch Beiträge, die sich mit der Gesundheit vulnerabler Gruppen auseinandersetzen.
	Qualifikation und Aus-, Fort- und Weiterbildung (ÖGD-Querschnittsthema)	QF	Kooperationen, die sich mit der Aus- Fort- und Weiterbildung von Fachpersonal im ÖGD auseinandersetzen.
	Methodenentwicklung (ÖGD-Querschnittsthema)	M	Kooperationen, die sich der Beschreibung, (Weiter-)entwicklung, Pilotierung oder Evaluation neuer methodischer Vorgehensweisen widmen.
	Qualitätssicherung (ÖGD-Querschnittsthema)	QS	Kooperationen, die sich mit der Sicherstellung einer hohen Struktur-, Prozess- und Ergebnisqualität im ÖGD beschäftigen.
	Schnittstelle Wissenschaft/Praxis als Forschungsinhalt (ÖGD-Querschnittsthema)	WP	Kooperationen, die sich inhaltlich mit der Schnittstelle zwischen Wissenschaft und Praxis im ÖGD (z.B. bzgl. Chancen und Hindernissen der Zusammenarbeit) auseinandersetzen.
	nicht eindeutig zuzuordnen/unklar	X	Kooperationen, die keiner anderen Kategorie zugeordnet werden könnten. Wird auch codiert, wenn der Eindruck vorliegt, dass eine weitere Kategorie vergeben werden sollte, das aber nicht klar benannt werden kann.
**Bundesländer**	-	_Bundesland_	Bundesländer in Deutschland in denen die Kooperation umgesetzt wurde/wird; Trennung der Bundesländer durch ein Semikolon + Leerzeichen
**Beschreibung (kurz)**	-	_Text_	Eigene möglichst prägnante und kurze Beschreibung der Kooperation (Darf am Abstract angelehnt sein, sollte aber möglichst kurz und knapp formuliert sein)

**Abstract/Volltext/Beschreibung**	-	_Text_	Wortwörtlicher Übertrag des Original-Abstracts oder der Original Beschreibung der Kooperation (Keine Änderungen vornehmen; 1:1 kopieren (Strg+C und Strg+V))
**Originalbeitrag**	-	_URL_	Link zum Originalbeitrag (Titel des Beitrags und Titel der unter der URL verfügbaren Quelle sollten übereinstimmen)
**Zusätzlicher Link I**	-	_URL_	Link zu weiteren für die Kooperation relevanten Inhalten
**Zusätzlicher Link II**	-	_URL_	Link zu weiteren für die Kooperation relevanten Inhalten

ZOM 3: Ausgefüllte PRISMA-ScR-Guideline (Preferred Reporting Items for Systematic reviews and Meta-Analyses extension for Scoping Reviews) Checkliste

SECTION	ITEM	PRISMA-ScR CHECKLIST ITEM	REPORTED ON PAGE #
**TITLE**			
Title	1	Identify the report as a scoping review.	i
**ABSTRACT**			
Structured summary	2	Provide a structured summary that includes (as applicable): background, objectives, eligibility criteria, sources of evidence, charting methods, results, and conclusions that relate to the review questions and objectives.	i-ii
**INTRODUCTION**			
Rationale	3	Describe the rationale for the review in the context of what is already known. Explain why the review questions/objectives lend themselves to a scoping review approach.	1
Objectives	4	Provide an explicit statement of the questions and objectives being addressed with reference to their key elements (e.g., population or participants, concepts, and context) or other relevant key elements used to conceptualize the review questions and/or objectives.	1
**METHODS**			
Protocol and registration	5	Indicate whether a review protocol exists; state if and where it can be accessed (e.g., a Web address); and if available, provide registration information, including the registration number.	9
Eligibility criteria	6	Specify characteristics of the sources of evidence used as eligibility criteria (e.g., years considered, language, and publication status), and provide a rationale.	2
Information sources*	7	Describe all information sources in the search (e.g., databases with dates of coverage and contact with authors to identify additional sources), as well as the date the most recent search was executed.	2-3
Search	8	Present the full electronic search strategy for at least 1 database, including any limits used, such that it could be repeated.	ZOM-1
Selection of sources of evidence†	9	State the process for selecting sources of evidence (i.e., screening and eligibility) included in the scoping review.	2-3
Data charting process‡	10	Describe the methods of charting data from the included sources of evidence (e.g., calibrated forms or forms that have been tested by the team before their use, and whether data charting was done independently or in duplicate) and any processes for obtaining and confirming data from investigators.	3
Data items	11	List and define all variables for which data were sought and any assumptions and simplifications made.	ZOM-2
Critical appraisal of individual sources of evidence§	12	If done, provide a rationale for conducting a critical appraisal of included sources of evidence; describe the methods used and how this information was used in any data synthesis (if appropriate).	n.a.
Synthesis of results	13	Describe the methods of handling and summarizing the data that were charted.	3

SECTION	ITEM	PRISMA-ScR CHECKLIST ITEM	REPORTED ON PAGE #
**RESULTS**			
Selection of sources of evidence	14	Give numbers of sources of evidence screened, assessed for eligibility, and included in the review, with reasons for exclusions at each stage, ideally using a flow diagram.	3
Characteristics of sources of evidence	15	For each source of evidence, present characteristics for which data were charted and provide the citations.	n.a.
Critical appraisal within sources of evidence	16	If done, present data on critical appraisal of included sources of evidence (see item 12).	n.a.
Results of individual sources of evidence	17	For each included source of evidence, present the relevant data that were charted that relate to the review questions and objectives.	n.a.
Synthesis of results	18	Summarize and/or present the charting results as they relate to the review questions and objectives.	4-5
**DISCUSSION**			
Summary of evidence	19	Summarize the main results (including an overview of concepts, themes, and types of evidence available), link to the review questions and objectives, and consider the relevance to key groups.	6-7
Limitations	20	Discuss the limitations of the scoping review process.	7-8
Conclusions	21	Provide a general interpretation of the results with respect to the review questions and objectives, as well as potential implications and/or next steps.	8
**FUNDING**			
Funding	22	Describe sources of funding for the included sources of evidence, as well as sources of funding for the scoping review. Describe the role of the funders of the scoping review.	9

JBI = Joanna Briggs Institute; PRISMA-ScR = Preferred Reporting Items for Systematic reviews and Meta-Analyses extension for Scoping Reviews.

* Where *sources of evidence* (see second footnote) are compiled from, such as bibliographic databases, social media platforms, and Web sites.

† A more inclusive/heterogeneous term used to account for the different types of evidence or data sources (e.g., quantitative and/or qualitative research, expert opinion, and policy documents) that may be eligible in a scoping review as opposed to only studies. This is not to be confused with *information sources* (see first footnote).

‡ The frameworks by Arksey and O'Malley (6) and Levac and colleagues (7) and the JBI guidance (4, 5) refer to the process of data extraction in a scoping review as data charting.

§ The process of systematically examining research evidence to assess its validity, results, and relevance before using it to inform a decision. This term is used for items 12 and 19 instead of "risk of bias" (which is more applicable to systematic reviews of interventions) to include and acknowledge the various sources of evidence that may be used in a scoping review (e.g., quantitative and/or qualitative research, expert opinion, and policy document). *From:* Tricco AC, Lillie E, Zarin W, O'Brien KK, Colquhoun H, Levac D, et al. PRISMA Extension for Scoping Reviews (PRISMAScR): Checklist and Explanation. Ann Intern Med. 2018;169:467–473. doi: 10.7326/M18-0850.

ZOM 4: Ausführliche Ergebnistabelle des Kooperationsscreenings

Auswertungsparameter	Definition	Fragestellung	Unterkategorien	Anzahl	Anteil[*1]
**Jahr**	Jahr, in welchem die Kooperation stattfand, bzw. bei mehrjährigen Kooperationen, in welchem Jahr sie startete	*Wie viele Kooperationen wurden pro Jahr identifiziert?*	vor 2015[*6]	19	3,1%
			2015	37	6,1%
			2016	42	6,9%
			2017	61	10,1%
			2018	56	9,3%
			2019	65	10,7%
			2020	42	6,9%
			2021	81	13,4%
			2022	57	9,4%
			2023	63	10,4%
			2024	82	13,6%
			**Gesamt[*5]**	**605**	
			k.a.	6	
**Kooperationsdauer**	Dauer der Kooperation in Jahren, wobei Kooperationsbeginn und -abschluss jeweils als volles Jahr gewertet werden	*Über welche Dauer erstrecken sich wie viele Kooperationen?*	≤ 1 Jahr	28	13,3%
			2-5 Jahre	112	53,3%
			6-10 Jahre	42	20,0%
			>10 Jahre	28	13,3%
			**Gesamt[*5]**	**210**	
			k.A.	401	
**Kooperationsstatus**	Angabe über den Status der Kooperation zum Stichtag der Analyse (30.03.2025)	*Welchem Status lassen sich wie viele Kooperationen zuordnen?*	Abgeschlossen	117	63,2%
			Laufend	68	36,8%
			**Gesamt[*5]**	**185**	
			k.A.	426	
**Kooperationsform**	Beschreibt die Art und Intensität der Zusammenarbeit	*Welche Kooperationsformen treten wie häufig auf?*	Kooperationen im Rahmen einer gemeinsamen Veranstaltung: zeitlich begrenzte Kooperationen, die ausschließlich auf die Planung und Durchführung einer spezifischen einmaligen Veranstaltung (z.B. Kongressvorträge oder Workshops) ausgerichtet sind.	160	26,2%

			Zusammenarbeit im Rahmen einer gemeinsamen Veröffentlichung: zeitlich begrenzte Kooperationen, deren Ergebnisse sowohl in klassischen Publikationen als auch in grauer Literatur veröffentlicht werden.	268	43,9%
			Kooperationen im Rahmen gemeinsamer Projekte: zeitlich begrenzte, zielorientierte Zusammenarbeit im Rahmen eines Projekts, die mit dem Abschluss des Projektzeitraums oder nach Erreichen der Meilensteine endet. Sie gliedern sich oft in Arbeitspakete, können langfristige Kooperationen initiieren und beinhalten häufig Veröffentlichungen sowie mindestens einen gemeinsamen Projektbericht.	126	20,6%
			Zeitlich (zunächst) unbegrenzte Kooperationen: bestehen langfristig ohne eine festgelegte Laufzeit bzw. ohne ein definiertes Enddatum und langfristig, werden aber oft nur zu bestimmten Anlässen oder in bestimmten Zyklen aktiv (z.B. Netzwerke, Forschungsverbünde; Fach- oder Arbeitsgruppen)	57	9,3%
			**Gesamt**[*5]	**611**	
**Publikationsform**	Angabe über die Kommunikations- und Veröffentlichungsformen der Kooperation	*Welche Formen der Veröffentlichung von Kooperationen treten wie häufig auf?*	Journal Article	161	27,6%
			Vortrag	159	27,3%
			Website	88	15,1%
			Poster(-präsentation)	81	13,9%
			Workshop	34	5,8%
			Fachforum / Symposium	18	3,1%
			Bericht	17	2,9%
			Podiumsdiskussion	4	0,7%
			Seminar	3	0,5%
			Abschlussarbeit	3	0,5%
			Stellungnahme	3	0,5%
			Arbeitsgruppe	2	0,3%
			Sonstiges	10	1,7%
			**Gesamt**[*5]	**583**	
			k.A.	28	

**Koordination / Erstautor:innenschaft**[*2]	Affiliation der koordinierenden Person bzw., bei Publikationen der / des Erstautors / der Erstautorin	*Wie häufig liegt die Koordination / Erstautor:innenschaft der Kooperationen bei Personen mit welcher Affiliation?*	Wissenschaft	342	57,7%
			ÖGD - Kommunale Ebene	139	23,4%
			ÖGD - Landesebene	117	19,7%
			ÖGD - Bundesebene	37	6,2%
			Sonstiges	8	1,3%
			**Gesamt**[*5]	**593**	
			k.A.	18	
**Co-Autor:innen / weitere beteiligte Personen**	Erfasst die Anzahl der über den/die Erstautor:in hinaus beteiligten Personen	*Wie viele weitere Personen sind an den Kooperationen beteiligt?*	0	40	6,9%
			1	53	9,2%
			2	66	11,4%
			3 bis 5	201	34,8%
			6 bis 10	160	27,7%
			≥ 10	57	9,9%
			**Gesamt**[*5]	**577**	
			k.a.	34	
**ÖGD-Ebene der Institutionen**[*4]	Verwaltungsebene (kommunal, Land, Bund), auf der die beteiligten ÖGD - Institutionen verortet sind	*Wie häufig sind Institutionen von welchen Verwaltungsebenen des ÖGD (Bund, Land, Kommune) an Kooperationen beteiligt?*	Kommunale Ebene	439	73,3%
			Landesebene	256	42,7%
			Bundesebene	72	12,0%
			**Gesamt**[*5]	**599**	
			k.A.	12	
**Umsetzungsebene**[*4]	Verwaltungsebene (kommunal, Land, Bund), auf der die Kooperation umgesetzt wird	*Wie häufig findet die Umsetzung der Kooperationen auf welcher Verwaltungsebene statt?*	Kommunale Ebene	355	68,7%
			Landesebene	127	24,6%
			Bundesebene	85	16,4%
			**Gesamt**[*5]	**517**	
			k.A.	94	
**Thematische Einordnung**	Inhaltliche Ausrichtung der Kooperation	*Welche ÖGD-relevanten Themengebiete und Arbeitsbereiche sind wie häufig Inhalt von Kooperationen?*	Gesundheitsförderung und Prävention (ÖGD-Arbeitsbereich)	162	26,5%
			Infektionsschutz & Hygiene (ÖGD-Arbeitsbereich)	146	23,9%
			Kinder- und Jugendgesundheit (ÖGD-Arbeitsbereich)	129	21,1%
			Epidemiologie (ÖGD-Arbeitsbereich)	106	17,3%

			Gesundheitsberichterstattung, Planung und Steuerung (ÖGD-Arbeitsbereich)	97	15,9%
			Soziale Ungleichheit (ÖGD-Arbeitsbereich)	54	8,8%
			Qualifikation und Aus-, Fort- und Weiterbildung (ÖGD-Querschnittsthema)	43	7,0%
			Schnittstelle Wissenschaft/Praxis als Forschungsinhalt (ÖGD-Querschnittsthema)	41	6,7%
			Umweltmedizin (ÖGD-Arbeitsbereich)	39	6,4%
			Sozialpsychiatrie/Mentale Gesundheit (ÖGD-Arbeitsbereich)	27	4,4%
			Methodenentwicklung (ÖGD-Querschnittsthema)	26	4,3%
			Migration und Gesundheit (ÖGD-Arbeitsbereich)	21	3,4%
			Qualitätssicherung (ÖGD-Querschnittsthema)	21	3,4%
			Zahnmedizin (ÖGD-Arbeitsbereich)	10	1,6%
			Sozialpharmazie (ÖGD-Arbeitsbereich)	7	1,1%
			Krisenmanagement (ÖGD-Arbeitsbereich)	5	0,8%
			Amtsärztlicher Dienst / Gutachtenwesen (ÖGD-Arbeitsbereich)	3	0,5%
			nicht eindeutig zuzuordnen	44	7,2%
			**Gesamt*5**	**611**	
**Bundesländer*4**	Bundesland in dem die Kooperation umgesetzt wird	*In welchem Bundesland werden wie häufig Kooperationen umgesetzt?*	Nordrhein-Westfalen	97	24,9%
			Baden-Württemberg	88	22,6%
			Bayern	82	21,0%
			Hessen	57	14,6%
			Niedersachsen	36	9,2%
			Berlin	33	8,5%
			Hamburg	23	5,9%
			Bremen	15	3,8%
			Sachsen	15	3,8%
			Rheinland-Pfalz	13	3,3%
			Schleswig-Holstein	13	3,3%
			Brandenburg	7	1,8%

			Mecklenburg-Vorpommern	6	1,5%
			Sachsen-Anhalt	5	1,3%
			Saarland	4	1,0%
			Thüringen	4	1,0%
			**Gesamt**[*5]	**390**	
			k.A.	221	
**Kooperierende Institutionen**[*4]	Institutionsform der an den Kooperationen beteiligten Akteure; jede Institution wurde einer Kategorie zugeordnet	*Wie häufig sind Institutionen welcher Institutionsformen in Kooperationen involviert gewesen?*	Universitäten, Hochschulen, Akademien und Institute	1039	33,0%[*3]
			Kommunalverwaltung	837	26,6%[*3]
			Landesverwaltung	301	9,6%[*3]
			Gesundheitseinrichtungen	252	8,0%[*3]
			Nicht-universitäre Forschungseinrichtungen und -institute	246	7,8%[*3]
			Fachgesellschaften und Netzwerke	95	3,0%[*3]
			Berufsverbände und -vertretungen	44	1,4%[*3]
			Krankenkassen	41	1,3%[*3]
			Bundesverwaltung	38	1,2%[*3]
			Wohlfahrtsverbände und Stiftungen	35	1,1%[*3]
			Privatwirtschaftliche Institutionen	19	0,6%[*3]
			Sonstige Institutionen	202	6,4%[*3]
			**Gesamt**	**3149**	

[*1] Bezieht sich auf die gültigen Kooperationen in der jeweiligen Kategorie, wobei die k.A.-Angaben rausgerechnet werden
[*2] Mehrfachaffiliationen sind möglich, wenn Personen gleichzeitig für Institutionen aus Praxis und Wissenschaft tätig sind
[*3] Die prozentualen Angaben beziehen sich auf den Anteil der jeweiligen Institutionsform an der Gesamtzahl der erfassten institutionellen Beteiligungen (N=3.149)
[*4] Mehrfachangaben möglich
[*5] Die Gesamtzahl berichtet alle gültigen Fälle (=Gesamtzahl der Kooperationen abzüglich der k.A.-Angaben)
[*6] Kooperationen, deren Publikationsjahr vor 2015 lag, deren Projektlaufzeit sich aber bis in den Zeitraum 2015-2024 streckt
